# Human orbital and anterior medial prefrontal cortex: Intrinsic connectivity parcellation and functional organization

**DOI:** 10.1007/s00429-017-1378-2

**Published:** 2017-03-02

**Authors:** Zoe Samara, Elisabeth A. T. Evers, Alexandros Goulas, Harry B. M. Uylings, Grazyna Rajkowska, Johannes G. Ramaekers, Peter Stiers

**Affiliations:** 1 Department of Neuropsychology and Psychopharmacology, Maastricht University, Universiteitssingel 40 (East), 6229 ER Maastricht, The Netherlands; 2 Max Planck Institute for Human Cognitive and Brain Sciences, Max Planck Research Group: Neuroanatomy and Connectivity, Stephanstrasse 1a, 04103 Leipzig, Germany; 3 Department of Anatomy and Neuroscience, Graduate School Neurosciences Amsterdam, VU University Medical Center, 1007 MB Amsterdam, The Netherlands; 4 Department of Psychiatry and Human Behavior, University of Mississippi Medical Center, Jackson, MS 39216-4505 USA

**Keywords:** Functional connectivity, Human, Orbital-medial prefrontal cortex, Modularity, MRI, Parcellation

## Abstract

The orbital and medial prefrontal cortex (OMPFC) has been implicated in decision-making, reward and emotion processing, and psychopathology, such as depression and obsessive–compulsive disorder. Human and monkey anatomical studies indicate the presence of various cortical subdivisions and suggest that these are organized in two extended networks, a medial and an orbital one. Attempts have been made to replicate these neuroanatomical findings in vivo using MRI techniques for imaging connectivity. These revealed several consistencies, but also many inconsistencies between reported results. Here, we use fMRI resting-state functional connectivity (FC) and data-driven modularity optimization to parcellate the OMPFC to investigate replicability of in vivo parcellation more systematically. By collecting two resting-state data sets per participant, we were able to quantify the reliability of the observed modules and their boundaries. Results show that there was significantly more than chance overlap in modules and their boundaries at the level of individual data sets. Moreover, some of these consistent boundaries significantly co-localized across participants. Hierarchical clustering showed that the whole-brain FC profiles of the OMPFC subregions separate them in two networks, a medial and orbital one, which overlap with the organization proposed by Barbas and Pandya (J Comp Neurol 286:353–375, 1989) and Ongür and Price (Cereb Cortex 10:206–219, 2000). We conclude that in vivo resting-state FC can delineate reliable and neuroanatomically plausible subdivisions that agree with established cytoarchitectonic trends and connectivity patterns, while other subdivisions do not show the same consistency across data sets and studies.

**Electronic supplementary material:**

The online version of this article (doi:10.1007/s00429-017-1378-2) contains supplementary material, which is available to authorized users.

## Introduction

The orbital and anterior medial part of the prefrontal cortex (OMPFC) has been implicated in goal-directed decision-making, reward representation, and emotional processing (Kringelbach 2005; Rolls 2016; Rolls and Grabenhorst 2008; Rushworth et al. 2007). The behavioural contribution of OMPFC relies on complex direct and indirect interactions between the specialized neuroanatomical units that constitute this part of cortex. Cytoarchitectonic studies in monkeys and humans have revealed the existence of several anatomical subdivisions within the OMPFC (Brodmann 1909; Carmichael and Price 1994; Petrides and Pandya 1994; Öngür et al. 2003; Mackey and Petrides 2009; Uylings et al. 2010) and tracing studies in the macaque have identified unique patterns of connectivity for each of these cortical fields (Cavada et al. 2000; Yeterian et al. 2012). Moreover, an additional, higher-level organization of the OMPFC has been proposed in the rhesus monkey. Based on cytoarchitectonic data, Barbas and Pandya (1989) distinguished a mediodorsal and a basoventral trend, coursing through the medial and orbital surface of the PFC, respectively. Similarly, using data from tracer studies, Carmichael and Price (1996) and Ongür and Price (2000) were able to establish two distinctive networks, each consisting of tightly interconnected subregions and characterized by a distinct pattern of cortico-cortical connections and couplings with limbic, autonomic, and other subcortical structures (Öngür and Price 2000). Based on its patterns of connectivity with the rest of the brain, the “orbital” network was thought to be a sensory-related system involved in integrating multi-modal stimuli, whereas the “medial” network was conceived as an output system involved in modulating the expression of emotion and action (Price and Drevets 2010).

These anatomical principles of organization are in agreement with recent insights into the involvement of different parts of OMPFC in affective decision-making. Cell recording and imaging studies indicate that the orbital cortex is responsible for the representation and updating of stimuli and their associated (primary and abstract) reward and affective values (e.g., Rushworth et al. 2011; Kringelbach 2005; Kringelbach and Rolls 2004; Murray 2007), whereas the medial cortex is particularly involved in goal-directed evaluations concerning action utilities (Rushworth et al. 2013). These insights are important for understanding neurological disorders. For instance, it has since long been known that structural damage to the OMPFC results in serious alternations in goal-oriented decision-making and affective planning (Bechara et al. 2000; Floden et al. 2008). Moreover, studies indicate that particularly the medial prefrontal network is implicated in mood disorders such as major depressive disorder (Price and Drevets 2010) and obsessive compulsive disorder (Norman et al. 2016).

While the anatomical organization and associated parcellation in subregions of the OMPFC have been firmly established in animal studies, it would be of great advantage for the study of OMPFC functioning in healthy persons and patients if this organization could be delineated in vivo in individual subjects. In the last decade, several studies have explored the possibilities of MRI techniques to parcellate the cerebral cortex into functionally meaningful cortical fields. The techniques used are diffusion weighted imaging (DWI)-based probabilistic tractography and resting-state fMRI-based functional connectivity (FC) analysis. The approach exploits the insight that functionally homogeneous cortical fields feature a unique pattern of anatomical connections (Krubitzer 1995; Passingham et al. 2002), which provide their neurons with the required afferent input and send computational output to the appropriate locations. Accordingly, Cohen et al. (2008) showed the existence of abrupt changes in rsFC profiles in a spatial array of cortical vertices. Independent of any prior information about a region’s function or topography, these consistent edges matched in size and number known cytoarchitectonic boundaries between cortical fields.

The first attempts to parcellate subparts of the OMPFC used DWI probabilistic tractography and focused on the subgenual medial PFC (Johansen-Berg et al. 2008), the cingulate gyrus (Beckmann et al. 2009), and recently, the entire orbital and medial frontal cortex (Neubert et al. 2015). Across these studies, some parcellation boundaries appear consistent, such as the delineation in the cingulate cortex of subgenual section, a division at the level of the genu of the corpus callosum, and the demarcation in cingulate cortex of dorsal anterior section from a midsection. However, a clustering analysis based on the whole-brain functional coupling of the delineated regions could not fully confirm segregation into a medial and an orbital network (Neubert et al. 2015, see their Supplementary Information). Orbital frontal cortex parcellation was also studied using resting-state FC data (Kahnt et al. 2012; Yeo et al. 2011). Despite variability in extent and number of orbital regions, the FC studies seem to converge with the Neubert et al. (2015)’s DWI study on a three-way division of the orbital surface in a lateral, middle, and medial sections. This division agrees with recent cytoarchitectonic findings (Uylings et al. 2010) and with evidence from functional activation studies (Berridge and Kringelbach 2015).

In addition to similarities, there is considerable variability across studies within as well as between MRI modalities, but also within and between cytoarchitectonic studies. This variability at least in part reflects the fundamental problem of scaling—i.e., different ways of meaningful integration and segregation of units exists depending on the scale of study, ranging from the macro level (whole-brain networks) to the micro level (cortical columns). For example, at the cytoarchitectonic level, BA 17 is defined by homogenous features, such as the stria of Gennari, while at the connectivity level, it is equally meaningfully subdivided into a foveal and peripheral visual field part (e.g., Buckner and Yeo 2014). The variability may also reflect replicability and reliability issues, however—issues that have not yet been systematically addressed.

In this study, we want to consider this replicability, in a new attempt to parcellate the orbital and anterior medial PFC. We will use a different imaging modality than Neubert et al. (2015)—resting-state FC instead of DWI—and a different parcellation method—Graph Theory-based module detection instead of *k*-means clustering. We will investigate whether subdivisions and module boundaries can be replicated in the same individuals and across individuals, and whether they agree with boundaries reported in other studies, such as Neubert et al. (2015). Moreover, we will ask whether the functional segregation of this large piece of cortex adheres to the differentiation of a medial and an orbital network, as established in the macaque monkey with invasive techniques (Carmichael and Price 1996; Ongür and Price 2000).

The methods employed here deviate from those in prior FC parcellation studies of OFC in two important ways. First, we follow Neubert et al. (2015) in performing the parcellations on the individual data. In contrast, Kahnt et al. (2012) and Yeo et al. (2011) averaged the FC data across participants prior to parcellation. While averaging avoids the problem of group-level integration, it also ignores the documented large inter-individual variability in gyrification (Chiavaras and Petrides 2000; Rodrigues et al. 2015) and size and positioning of cortical fields (Uylings et al. 2005). Moreover, it excludes the possibility to study replicability at the individual level. Second, we use a modularity detection algorithm (Meunier et al. 2009; Shen et al. 2010; De Meo et al. 2011), rather than a clustering method, to group voxels into modules. Clustering methods require the a priori specification of the number of modules, which impels researchers to perform the parcellation for a parametric range of numbers of modules and then pick a “best” solution. The graph theory-based approach adopted here aims to group voxels in a way that maximizes modularity, i.e., groups that exhibit more connections between one another than the ones expected by chance. Modularity in a set of voxels increases inversely with the initial connection density amongst them, up to the point where the voxels no longer constitute a fully connected set. We will, therefore, parcellate the voxel-to-voxel connection matrix at the lowest density that still preserves full connectedness.

Modularity maximization parcellation of resting-state data has been used previously to delineate subdivisions within the basal ganglia (Barnes et al. 2010) and within the lateral PFC (Goulas et al. 2012). Three important implications of modularity optimization have to be considered. First, modularity maximization is a nondeterministic polynomial time-complete problem, which in practice can only be solved approximately (Fortunato 2010). As a consequence, implementations involving such problems are stochastic, yielding somewhat different solutions for repeated analyses. It has also been shown for neuroimaging data that modularity maximization is a degenerate process, with a large number of near maximum modularity solutions (Good et al. 2010; Fortunato 2010; Rubinov and Sporns 2011). Second, the number of modules obtained is determined by the data and can vary within and between individuals. This will complicate the integration of individual parcellation schemes into a group scheme. Third, the connectivity matrix for each participant is based on intrinsic functional connectivity strength—i.e., the strength of functional coupling between the voxels within the parcellation region—instead of similarity of FC profiles of voxels with the rest of the brain. This is because graph modules are defined as groups of voxels with stronger than average interconnections. The reliance on intrinsic instead of extrinsic connectivity does not mean, however, that results will be more biased by local noise or vascular effects that boost the time course correlations of neighboring voxels. Because the same time courses are used in extrinsic as in intrinsic FC, such biases are equally strong in both cases: more similar time courses in nearby voxels will lead to more similar connectivity profiles with the rest of the brain as well as stronger functional coupling between them.

## **M**aterials and methods

### Participants

Thirty-four psychiatrically and neurologically healthy participants (21 females; mean age, 32.3 years; SD, 14.5 years) were subjected to one MRI scanning session after giving their informed consent. The scanning protocol included two resting-state scans of approximately 6.5 min with identical scanning parameters and instructions, separated approximately 10 min in time. Participants were instructed to fixate on a cross at the center of the screen, keep their eyes open, and refrain from intentionally engaging in specific mental tasks or falling asleep during the scan.

### fMRI acquisition

Scanning was conducted on a Siemens MAGNETOM Allegra 3T MRI head-only scanner. Head motion was constrained by the use of foam padding. For each participant, 153 T2*-weighted gradient echo planar images (EPI) with 41 slices were acquired (except for 6 participants for whom 203 images were available). EPI can suffer substantial loss of BOLD sensitivity and geometric distortions due to magnetic field inhomogeneity near air tissue interfaces. To minimize MRI signal loss and recover the true spatial signal positions in the OFC, we: (a) used an optimized echo time, (b) tilted the slices (~30° angle), and (c) generated a field map to offline correct susceptibility-related signal displacements. Imaging parameters for the resting-state sequence were as follows: TR, 2500 ms; TE, 25 ms; flip angle, 90°; matrix size, 128 × 96; and FOV, 256 mm; distance factor, 20%; resulting in a voxel size of 2 × 2 × 3 mm. The gradient echo image used to generate the field map had the same grid and slice orientation as the functional images (TR 704 ms; TE 5.11, 7.57 ms; flip angle 60°). To enable the localization of functional data, a high-resolution T1-weighted image was acquired with the following parameters: TR 2250 ms; TE 2.6 ms; flip angle 9°; FOV 256 mm; slice thickness 1 mm; matrix size 256 × 256; number of slices 192; voxel size 1 × 1 × 1 mm.

### fMRI preprocessing

Preprocessing of fMRI data was performed using the SPM 5 software (Welcome Trust Center for Neuroimaging, London, UK). The functional data were subjected to the following preprocessing: slice time correction, spatial correction using the field map, realignment, co-registration with the anatomical scan, normalization to the Montreal Neurological Institute (MNI) template (ICBM-152), reslicing to 3 mm isotropic voxels, and smoothing with a 6 mm full width half maximum (FWHM) Gaussian kernel. The T1-weighted images were segmented into grey matter, white matter, and cerebrospinal fluid tissue maps, and these maps were later used in the analyses. Furthermore, we removed non-neuronal contributions from the BOLD signal by regressing the following nuisance variables: the six volume realignment parameters, the average time series in white matter and CSF voxels, the session-specific mean, and the intrinsic autocorrelations. The global brain signal or average grey matter signal was not included as a regressor. Finally, the residual volumes of the multiple regression were Fourier band pass filtered (0.01–0.1 Hz).

Head motion has been shown to significantly underestimate long-range and overestimating short-range FC connectivity, even after regressing out volume to volume head motion measures (Power et al. 2012; van; Dijk et al. 2012). To further reduce this bias, we took the following approach: (1) we identified scans during which the frame-wise displacement exceeded 0.4 mm [13.3% of voxel size; i.e., translation in the z direction or rotation in the *x* direction corresponding to 0.4 mm z-displacement of frontopolar voxels, assuming an x-rotation point 88 mm from the frontal pole; Talairach and Tournoux (1988)], (2) we excluded the identified volumes together with the 1-back and 2-forward volumes [to avoid spin history assumptions’ violations caused by movement; Power et al. 2012)], and (3) we excluded participants for whom less than 120 volumes [i.e., 5 min; van Dijk et al. 2010)] of resting data remained after the correction (mean duration, 6.4 min; SD, 0.8 min).

### OMPFC intrinsic FC-based parcellation

The parcellation analysis was performed for each participant and each hemisphere separately. For each participant, the voxels selected for parcellation comprised all the voxels that fell both within the person’s normalized grey matter mask (density > 0.5) and a liberal OMPFC ROI mask (left or right hemisphere). Thus, the parcellation mask differed between participants to accommodate anatomical variation and avoid contaminating of the analysis with none-cortical voxels. The liberal ROI mask was constructed from the Automated Anatomical Labeling (AAL) map in MNI space (WFU PickAtlas; Maldjian et al. 2003; Rollset al. 2015; Tzourio-Mazoyer et al. 2002). It comprised left-side AAL regions with the following labels: “frontal superior orbital”, “frontal middle orbital”, “frontal inferior orbital”, “frontal medial orbital”, “rectus”, “cingulum anterior”, and “frontal superior medial”. From the region labeled “frontal superior medial”, only a part was included, extending dorsally until the horizontal border defined by the anterior cingulate AAL label (manually drawn using MRIcron; Rorden and Brett 2000). The ROI mask was expanded spatially to ensure coverage of grey matter in all participants and to cover also parts of areas boarding the orbital and medial areas of interest. The latter allow empirical delineation of the full extent of areas of interest at the boundary of the mask. The expansion of the mask comprised of a twice repeated 10 mm FWHM Gaussian smoothing followed by high-pass thresholding at 0.2 density.

For the voxels selected for parcellation, a voxel-by-voxel correlation matrix was constructed by computing the Pearson correlation between their cleaned time courses measured during the first resting-state scan. A high-pass absolute weight threshold was applied to the correlation matrix to eliminate the weak, less-significant links that most likely represent spurious connections (Rubinov and Sporns 2010). Because modularity is inversely related to graph density (Goulas et al. 2012) and since our aim was to retrieve the maximal modularity solution for grouping the voxels into modules, we searched for the lowest connection density that still yielded a connected graph. The connection densities investigated were: 0.25, 0.5, 1.0, 1.5, 2.0, 2.5, 3.0, 3.5, and 4.0%.

To partition a thresholded correlation matrix into discrete modules, we employed the Louvain module detection algorithm [Blondel et al. 2008; Brain Connectivity Toolbox (Rubinov and Sporns 2010)], one of the best performing algorithms for fast and efficient detection of modules in extended networks (Lancichinetti and Fortunato 2009). The modularity statistic quantifies how well a network can be subdivided into groups of nodes (voxels) with higher than chance connectivity in between them (Girvan and Newman 2002; Newman 2006). Applied to brain networks, it can be used to delineate neurobiological meaningful functional units (e.g., Goulas et al. 2012; Meunier et al. 2009; Rubinov and Sporns 2010). Modularity is defined as follows:

1 $$Q=~\underset{i=1}{\overset{k}{\mathop \sum }}\,\left[ \frac{{{e}_{i}}}{m}-~{{\left( \frac{{{d}_{i}}}{2m} \right)}^{2}} \right],$$

where *e*_*i*_ is the amount of edges (connections) linking nodes (voxels) within module *i, d*_*i*_ is the total amount of edges of module *i* nodes (i.e., degree of module *i*), and *m* is the total number of edges in the graph (i.e., network degree). Large *Q* values indicate the presence of community structure within the graph. To compensate for the stochastic nature of the algorithm, each parcellation analysis of individual data at a particular threshold was repeated 50 times and the solution with the highest *Q* value was selected as the final solution (Sporns et al. 2007). The parcellation procedure results in the unique classification of every voxel in the OMPFC into one of the modules in the solution.

To test the statistical significance of the observed modularity structure, we compared the obtained *Q* value with the *Q* value of null models computed from the individual data sets. Zalesky et al. (2012) have recently drawn attention to the fact that observations of brain networks that use correlation as a measure of connectivity are inherently more clustered than random networks. For this reason, they should be benchmarked against null networks that preserve the transitive structure of correlation networks. We created such null networks by applying the Hirschberger–Qi–Steuer algorithm (for algorithm and details see Zalesky et al. 2012) to the individual correlation matrices. This algorithm generates random null covariance matrices with distributional properties matched to the observed matrices. The resulting null covariance matrices were thresholded to match the density of the original matrices.

### Group-wise clustering

Modules obtained from parcellating individual data sets were grouped together to capture their individual transcending commonalities. Because individual parcellations differed in voxel space (within each participant the parcellation took place within his/her unique grey matter map) and had different numbers of modules, the grouping was based on the spatial proximity of the modules’ center of mass (COM), within the Euclidean coordinates of the MNI space. Spatial proximity was defined as the inverse of the Euclidean distance between the COM of modules from two different parcellations. The integration progressed iteratively. At each iteration, first, a cost matrix was computed for matching each parcellation with every other parcellation. The matching cost for a pair of parcellations was the sum of the distances between their assigned modules. Module assignment between pairs of parcellations was based on minimizing the matching cost using the modified Hungarian assignment algorithm (Munkres 1957; Cao 2008). In the second step, the pairs of parcellations with the lowest matching cost were merged by weighted averaging (for details, see Supplementary Information, section “Methods”, point 1), one after the other, and then eliminated from the cost matrix. The merged parcellations entered the next iteration level and the procedure was repeated until a final set of COMs was obtained. Each final COM represented a cluster of modules from individual parcellations merged into this common COM. Note that not all participants will be represented in every final cluster, due to different numbers of modules per participant/parcellation.

### Replicability of intrinsic FC parcellation

To investigate replicability of parcellation results, we analyzed for each participants a second, independently resting-state scan acquired in a different run during the same session. The within participant consistency of the obtained modules across the two data sets was compared first to the maximum possible consistency and second to chance consistency. This maximum possible consistency is less than 100% due to the stochastic nature of the modularity detection algorithm used. The maximum replicability was estimated by re-analysing the same data sets twice. To test whether the observed consistency exceeded the chance level, it was compared to the replicability between null models, in which a matching number of modules is positioned purely randomly. These models implement the null hypothesis that the spatial location of the modules is not dependent on information in the connectivity matrix. The creation details of these null models are described in Supplementary Information (“Methods”, point 2).

The measures used to compare parcellation similarity were the Dice similarity coefficient (Crum et al. 2006) and normalized mutual information. Both measures will be around 0.5 for completely mismatching parcellations and 1.0 for perfectly overlapping parcellations. The normalized mutual information has the advantage over the Dice similarity coefficient that it does not require an assignment of modules between the parcellations prior to quantification, which introduces additional room for error. The modified Hungarian assignment algorithm (Munkres 1957; Cao 2008) was used for modules assignment.

Replicability was further investigated by looking at the module boundaries. A boundary in a parcellation was defined in volume space as a center-surround discrepancy, with the center being a single voxel’s module identification number, and the voxel’s surround comprising the averaged value of any of the six voxels touching it on each of its sides. Voxels outside of the parcellation patch were not considered (i.e., surround < six voxels) to prevent voxels on the edge of the parcellation patch from being counted as boundary voxels. For each pair of parcellations, the consistency of boundary voxels was quantified by the number of voxels that were identified as boundary voxel in both parcellations relative to the number of boundary voxels in each parcellation, expressed as a Dice coefficient.

Replicability of boundaries was also assessed at the group level, by counting the incidence of voxels being boundary voxels across participants. This requires summation across participants in voxel space, which can only yield a rough approximation of boundary co-localization. To improve co-localization, consistent boundary images per participant and the similar images derived from the random module models were smoothed with a 6 mm FWHM Gaussian kernel. A voxel-wise *t*-test of the observed sum of co-localized boundaries against the co-localization of boundaries in the random models was performed and differences evaluated at the alpha level of 0.05, FDR-level correction for multiple comparison (Genovese et al. 2002).

Finally, group-wise replicability was also evaluated from a visual comparison of different group-wise parcellation results. First, the group-integrated parcellation of the left hemisphere was compared to that from the right hemisphere. Therefore, the connectivity-based parcellation and group-wise clustering described above were also applied to the right OMPFC of the same resting-state run. In addition, we tested the replicability within the left hemisphere across runs, by applying the group-wise clustering also to the parcellation results from the left OMPFC of the second resting-state run in the scanning session. We compared the similarity of the group-wise clustering result between each hemisphere and run by plotting their spatial extent to allow visual inspecting of their correspondence with our main results.

### OFC signal coverage and inter-run correlation stability

EPI suffers substantial loss of BOLD sensitivity near air tissue interfaces and FC metrics are sensitive to the levels of signal amplitude and signal-to-noise ratio (Golestani and Goodyear 2011).

To quantify the severity of signal dropout in the artifact-susceptible areas, we calculated the relative signal intensity, which is the signal intensity of each voxel (averaged across time) relative to the mean signal intensity of all grey matter voxels (Smits et al. 2007). In addition, we computed the signal-to-noise ratio of the time series (tSNR), defined as the mean intensity of every voxel in the time series divided by its standard deviation across time (Triantafyllou et al. 2011; Golestani and Goodyear 2011).

To assess the impact of signal quality loss on the connectivity metric used, we estimated the stability of the whole-brain FC profiles of each OMPFC voxel across the two separate resting-state runs, using the eta^2^ coefficient:

2 $$\text{et}{{\text{a}}^{2}}=1-~\frac{S{{S}_{\text{Within}}}}{S{{S}_{\text{Total}}}}=1-~\frac{\mathop{\sum }_{i=1}^{n}\left[ {{\left( {{a}_{i~}}-~{{m}_{i}} \right)}^{2}}+~{{({{b}_{i}}-{{m}_{i}}~)}^{2}} \right]}{\mathop{\sum }_{i=1}^{n}[{{({{a}_{i}}-M)}^{2}}+~{{({{b}_{i}}-M)}^{2}}]},$$

where *a*_*i*_ and *b*_*i*_ are the correlations of voxel *i* in the first run and second run, respectively, *m*_*i*_ is the mean correlation of both runs at position *i*, and *M* is the grand mean of correlations across all locations in both runs. The eta^2^ coefficient varies from 0 (no similarity) to 1 (identical) and directly quantifies the difference in the values of the same voxel in the two runs (Cohen et al. 2008). A general linear mixed model analysis was used to test whether the inter-run stability differed significantly between the individual clusters by running. The model used heterogeneous compound symmetry as covariance structure and cluster as a fixed effects factor. The estimation method was restricted maximum-likelihood estimation (maximum iterations = 150). The advantage of general linear mixed models is that they allow the analysis of repeated-measures data in unbalanced designs, as is the case here, since every participant has a module in many but not all group-level clusters of modules. To control the family wise error rate, we used Holm’s sequential rejective Bonferroni correction (Holm 1979; Holland and Copenhaver 1987). All pairwise comparisons among means were adjusted to a corrected alpha of 0.05.

To test whether tSNR had a significant effect on the inter-run correlation stability, we ran the general linear model with the SPSS procedure mixed again this time using tSNR as a covariate. The amount of variance explained by tSNR was calculated as follows:

3 $${{r}^{2}}=1-~\frac{{{{\hat{\sigma }}}^{2}}}{\hat{\sigma }_{0}^{2}},$$

where *r* ^2^ is the proportion of the variance explained, *σ* is the standard error estimate in the model with tSNR as predictor, and *σ*_0_ is the standard error estimate of the null model.

### Similarity of whole-brain FC profiles of the OMPFC subdivisions

For every cluster, a whole-brain FC profile was created by placing a spherical seed of 4 mm radius at the COM of each participant’s module and calculating Pearson’s correlation coefficient between the average seed voxels time course and the time course of every voxels in the rest of the brain. The spherical seed approach was chosen over using the entire module as seed to get a maximal spatial spread of the seeds while avoiding contamination from adjacent modules. This procedure resulted in whole-brain *r* maps for every module at the individual level. The maps were subsequently r-to-Z transformed using Fisher’s formula. Cluster-wise functional connectivity maps was created by averaging over the r maps of all the modules that had been assigned to the particular cluster.

To examine whether our OMPFC subdivisions could be distinguished into spatially extended networks based in FC profile similarities, we used agglomerative hierarchical cluster analysis which does not involve *a priori* assumptions about the number of groups present in the data and outputs a “bottom-up” hierarchy of areas in the form of a dendrogram. First, we calculated the (dis)similarity matrix for the whole-brain connectivity profiles of all clusters using correlation as the similarity metric (1 − *r*) and subsequently created a dendrogram to represent the hierarchies in the data. To select the most appropriate linkage method for the construction of the dendrogram, we ran the analysis using the following linkage methods: centroid, average, single, median, complete, weighted, and Ward. For each of these methods, we computed the cophenetic correlation coefficient of the resulting dendrogram, a measure of how well the original distances in the data are represented. From these, we selected the linkage method that generated a dendrogram which contained clusters proceeding hierarchically (i.e., monotonic) and had the highest cophenetic coefficient.

## Results

### OMPFC intrinsic FC-based parcellation and group-wise clustering

We used the intrinsic FC of voxels within the OMPFC to parcellate individual cortical patches into modules with the Louvain module detection algorithm. Although we performed the parcellation analysis in both hemispheres using separate patch masks and obtained comparable partitions, the results reported in this paper center on the left hemisphere for practical reasons. Results for the right hemisphere are presented only in the section on the replicability of the parcellation analysis, below.

**Fig. 1:**
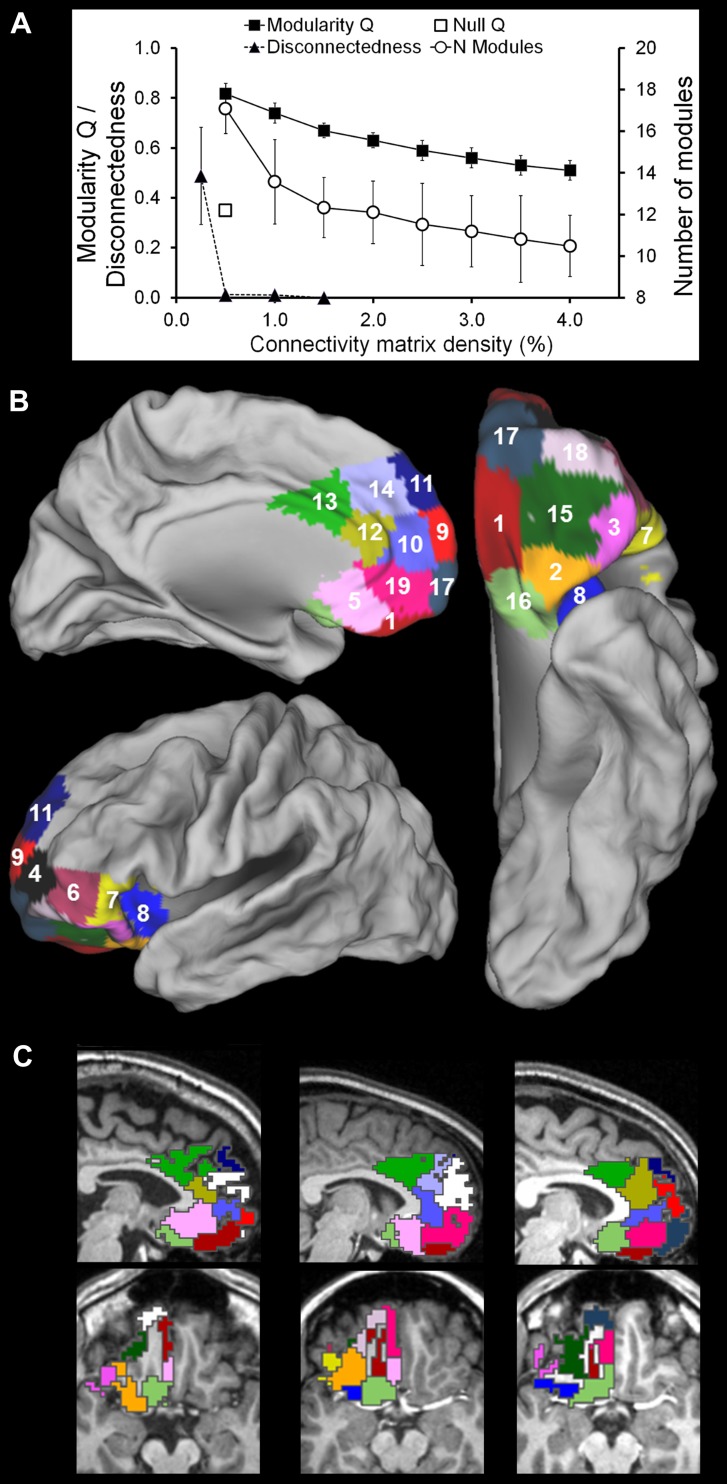
Parcellation of the orbital and medial prefrontal cortex at a connectivity matrix density of 0.5%. **a** Effect of matrix density in the range of 4.0 to 0.25% on graph connectedness, parcellation modularity, and number of modules found. At the lowest density level (0.25%), graph connectedness broke down. Hence, a density of 0.5% was chosen for the parcellation analysis. **b** Group-wise maximum probability map of clusters of parcellation modules in the* left* hemisphere; each cluster of modules was assigned a unique *number* and *color*. **c** Parcellation modules in three representative participants. Modules haven been given the *color* of the cluster of modules to which they were assigned in the group clustering presented in part B. Modules *colored white* were not assigned to any of the clusters

Figure 1a shows the effect that graph density had on the connectedness and the modularity of the graph. Reducing the connection density of the graph strongly improved its modularity. However, at a certain point, the number of connections became so low that the graph fell apart in disconnected parts. The proportion of nodes/voxels no longer connected with the rest of the graph only slightly increased from 1% (1.03 ± 1.01%) to 0.5% density (1.18 ± 1.14%), the difference being not statistically significant (*t*(20) = 1.27, *p* = 0.22). At the next lower density of 0.25%, however, connectedness broke down with 48.8 ± 19.5% voxels lost due to disconnection. Therefore, we here report on the modules obtained with a matrix density of 0.5%. The average modularity *Q* across participants at that density was 0.826 ± 0.024. These *Q* values are high, and well above 0.4 which is considered an indicator of a modular data structure (Fortunato 2010). Moreover, the observed *Q* values were significantly higher than the *Q* values of the null covariance models (*t*(33) = 41.73, *p* < 0.000). These results are consistent with previous studies using similar methods (Meunier et al. 2009; Barnes et al. 2010; Goulas et al. 2012).

The number of modules found was not set a priori, but determined by the modularity seeking algorithm based on the data. As a consequence, the number of modules found varied across participants, and ranged from 14 to 22 (mean 17.4 ± 1.8). Individual modules were grouped across participants into clusters. Because the number of modules varied per participant, not all participants were required to contribute to each clusters (see “Materials and Methods”, Section “Group-wise clustering”, and the Discussion for an elaboration on this issue). The group-wise clustering revealed the existence of 19 group-representative clusters of modules in the left hemisphere, comprising minimally modules from 22 participants (65% of sample size). Each cluster was assigned a unique identification number in an arbitrary way. An overview of the clusters of modules, their COMs, and their size can be found in Table 1.

**Table 1:** Cluster numbers correspond to numbering of parcellation map (Fig. 1)

Coordinates and size of the left hemisphere clusters								
Cluster	Center of Mass (COM)						Volume (mm^3^)	
	*X*		*Y*		*Z*		Mean	SD
	Mean	SD	Mean	SD	Mean	SD		
C01	−8.04	3.14	37.98	7.75	−25.26	2.59	3.43858	1.28234
C02	−26.38	3.24	23.99	5.64	−21.22	2.46	3.33072	1.28949
C03	−35.81	6.30	33.61	4.39	−15.52	2.16	2.91713	1.50444
C04	−27.07	4.07	59.64	2.43	−2.27	3.22	4.23203	1.35218
C05	−2.79	0.70	29.27	5.91	−14.09	3.00	4.31730	1.45231
C06	−44.65	1.98	45.85	2.85	−3.53	2.32	3.89250	1.40699
C07	−46.29	3.03	28.67	3.88	−5.48	3.47	4.13013	1.20204
C08	−35.20	3.36	20.49	1.74	−9.02	2.66	4.86000	1.33199
C09	−11.47	5.03	65.28	3.30	5.72	6.06	3.33161	1.42413
C10	−5.00	1.38	50.97	5.13	2.18	5.85	4.31348	1.43981
C11	−11.06	3.41	58.98	2.78	26.39	5.01	3.86361	1.29157
C12	−4.48	1.69	41.51	4.73	12.03	4.15	3.87700	1.19410
C13	−3.29	1.31	23.91	4.16	28.45	2.52	4.54191	1.36320
C14	−5.06	1.23	43.10	6.27	27.71	4.04	4.17246	1.22019
C15	−26.63	3.95	39.21	3.20	−18.05	2.73	4.84858	1.77168
C16	−14.54	4.62	15.11	2.79	−19.45	2.30	4.47525	1.73866
C17	−12.89	6.06	60.59	4.37	−13.84	4.47	3.44203	1.44843
C18	−30.25	7.15	53.54	4.71	−13.33	4.27	2.91109	1.48657
C19	−3.73	1.06	44.62	5.98	−11.24	5.15	4.53311	1.42786

Reported coordinates are in MNI space and volume information concerns all modules corresponding to each cluster

*SD* standard deviation

To give an impression of the location of the clusters, we created a voxel-wise maximum probability map of the clusters (Fig. 1b). It should be noted, however, that this is just one way of visualization and other integration methods might yield somewhat differently looking cortical subdivisions. Seven of the clusters, C01, C16, C02, C03, C15, C17, and C18, were located at the orbital surface of the hemisphere; clusters C06 and C04 occupy the ventrolateral PFC and clusters C07 and C08 were located on the insulo-opercular cortex. At the medial wall, clusters C05, C19, C12, and C13 were distributed rostrocaudally around the corpus callosum, covering the cingulate cortex with extensions beyond it. Clusters C10 and C14 cover the (superior or) para cingulate gyrus and the medial part of the superior frontal gyrus and clusters C09 and C11 occupy the dorsal part of the superior frontal gyrus. As mentioned above, not all participants have a module that corresponds to one (or more) clusters. Nonetheless, correspondence of the classified individual modules with the group clusters of the parcellation map is generally good as illustrated in Fig. 1c for three representative participants.

### Replicability of intrinsic FC parcellation

Given the considerable differences in available parcellation schemes for the orbital and medial PFC, a central question is that of the reliability of parcellation results—i.e., the similarity of modules when the analysis is repeated with the same data set, or with a different data set from the same individual, and this compared to when the modules are randomly positioned.

Due to the stochastic nature of the modularity maximization algorithm, each re-analysis of the same data set yields a somewhat different solution. The average normalized mutual information between two successive analyses of the same data was 0.933 ± 0.024. After matching the modules in the two parcellations, the proportion of commonly assigned voxels was 91.5 ± 3.8%, giving an average Dice Similarity coefficient of 0.908 ± 0.039. When only looking at the voxels marking boundaries between modules, boundary replicability expressed as a Dice similarity coefficient was 0.895 ± 0.041.

The spatial similarity of the module structure was significantly lower for all measures when parcellations from two different data sets of each participant were being compared [smallest *t*(66) = 22.7, *p* < 0.001]. Mutual information dropped to an average of 0.711 ± 0.036, and the Dice coefficient to 0.626 ± 0.061. For the replication of module boundaries, the Dice coefficient was 0.467 ± 0.050. However, these spatial similarity indicators were still higher than for random module models. The random models did not differ from their corresponding real parcellations in the number of modules, the size of the modules, or the distance to the nearest neighboring module [largest *t*(66) = 0.8, *p* = 0.217]. However, they scored significantly lower on all module similarity measures [smallest *t*(66) = 9.5, *p* < 0.001]. The normalized mutual information between the random model pairs was on average 0.641 ± 0.023, while the common voxels in matched modules was 50.2 ± 4.2%, yielding a Dice similarity coefficient of 0.497 ± 0.043. The Dice coefficient for co-localized module boundary voxels was only 0.360 ± 0.040. These results make clear that although the parcellation results only partially replicate across different data sets from the same participants, at least a significant portion of the spatial features is preserved.

**Fig. 2:**
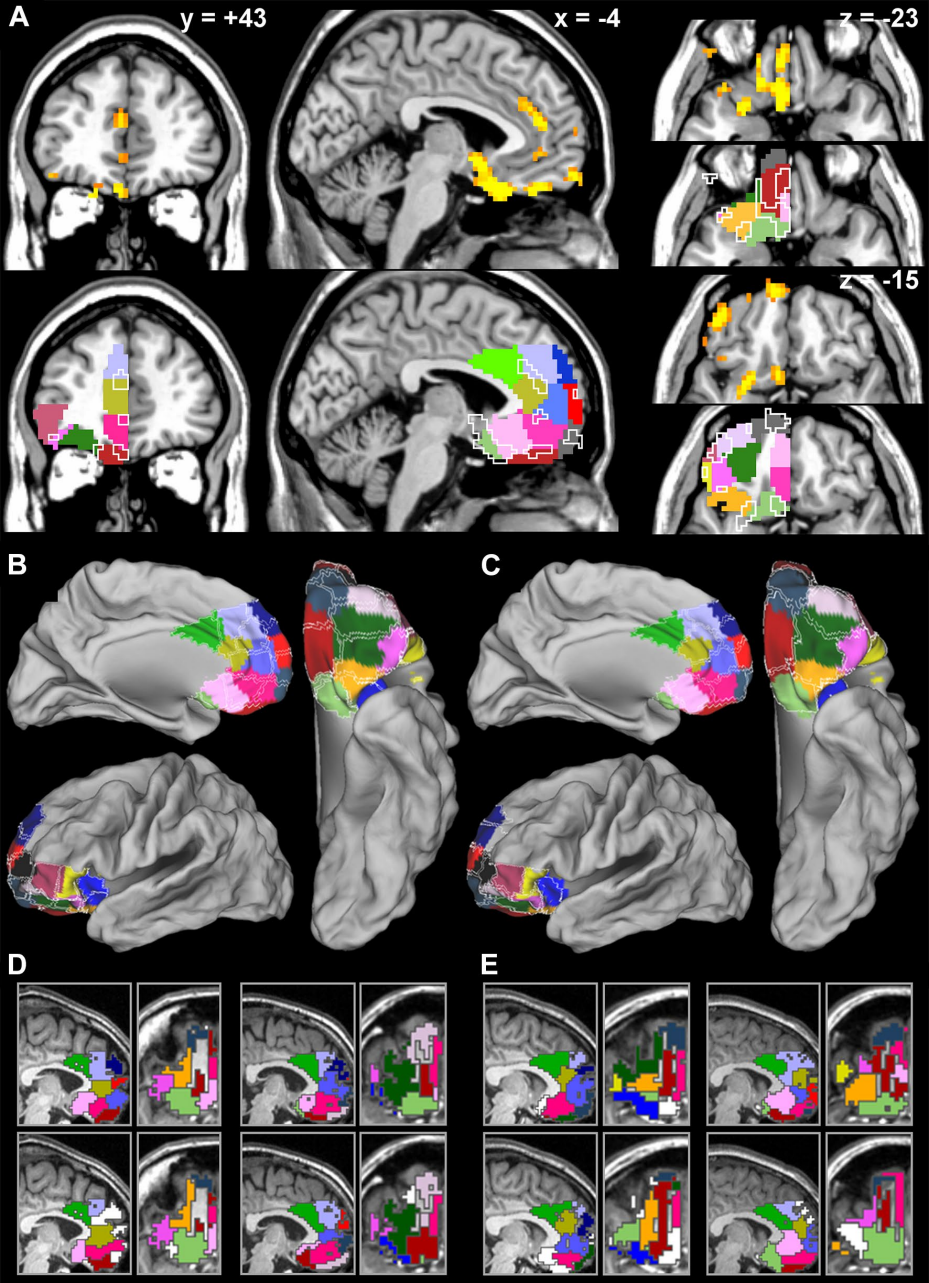
Replicability of parcellation results. **a** Co-localization of within participant reliable boundary voxels across participants. *Top row* shows voxels with significantly higher replicated boundary values compared to similar replicated boundary measures in null models of randomly located, size-matched modules. Thresholded at *t* = 3.04, *p* < 0.05 FDR corrected for multiple comparisons. Bottom row shows the same significant voxel clusters drawn as outlines over the group-wise maximum probability map of the parcellation clusters presented in Fig. 1, to show that mostly the consistently located boundaries overlap the transition between clusters in the maximum probability map. **b** Replicability of parcellation map across functional runs. Clusters in the* left* hemisphere of the first resting-state scan (Fig. 1) are plotted in *color* while the clusters in the* left* hemisphere of the second resting-state scan are overlaid as borders in *white*. **c** Replicability of parcellation map across hemispheres. Clusters in the* left* hemisphere of the first resting-state scan (Fig. 1) are plotted in *color*, while the clusters in the* right* hemisphere are overlaid as borders in white. **d** Replicability across runs at the level of individual participants. *Top panels* (medial wall *left*, orbital surface *right*) depict parcellation module maps in the* left* hemisphere of the first resting-state scan in two representative participants. *Color* coding corresponds to maximum probability cluster map presented in Fig. 1. Bottom panels show the parcellation module maps of the same participants in the* left* hemisphere of the second resting-state scan. White modules were not assigned to any of the clusters. **e** Replicability across hemispheres at the level of individual participants. *Top panels* (medial wall *left*, orbital surface *right*) depict parcellation module maps in the* left* hemisphere of the first resting-state scan in two representative participants. *Color* coding corresponds to maximum probability cluster map (Fig. 1). Bottom panels show the parcellation module maps of the same participants in the* right* hemisphere of the same scan. *White* modules were not assigned to any of the clusters

To investigate whether there was any consistency in the location of these persistent features across different participants, we performed a voxel-wise GLM analysis on the images that mapped the location of the replicated boundary voxels in each participant. To compensate for the considerable individual variability in grey matter anatomy, the images were smoothed with a 6 mm FWHM Gaussian kernel prior to entering the analysis. This analysis revealed several regions where boundaries significantly co-localized across participants (Fig. 2a, top row). These boundary regions tended to coincide with regions where cluster probabilities changed from one cluster to another (Fig. 2a bottom row). Significant co-localization of replicable boundaries was found medially in the dorsal aspect of the (para)cingulate sulcus, in the cortex anterior to the genu of the corpus callosum, and along the convexity between the orbital and the medial cortical surface. Orbitally, a boundary was found longitudinally between the olfactory and medial orbital sulci and another near the lateral orbital sulcus.

As a last step in investigating replicability, we computed similar maximum probability maps for the left hemisphere based on the second resting-state run and for the right hemisphere based on the first functional run (data from the same participants). To appreciate the consistency of solutions across runs and hemispheres, these maps are depicted in Fig. 2b and c, overlaid as boundary outlines on the maximum probability map of the main solution (left hemisphere, first resting-state run, as presented in Fig. 1; right hemisphere data are flipped in the left–right dimension). Using the right hemisphere as a replication test for the analysis of the left hemisphere, we do not want to imply that there are no functional or even structural interhemispheric differences. The comparison intended here is only valid at the macroscopic level, in terms of the number and general spatial lay-out of cortical fields. As can be expected from the partial replicability of parcellations at the individual level, discussed above, shifting boundaries can be observed in several subregions. Despite these local shifts, however, there is substantial agreement between these group-level maximum probability maps, particularly in those parts of OMPFC that emerged as regions of replicable boundaries within and across participants.

### OFC signal coverage and inter-run correlation stability

**Fig. 3:**
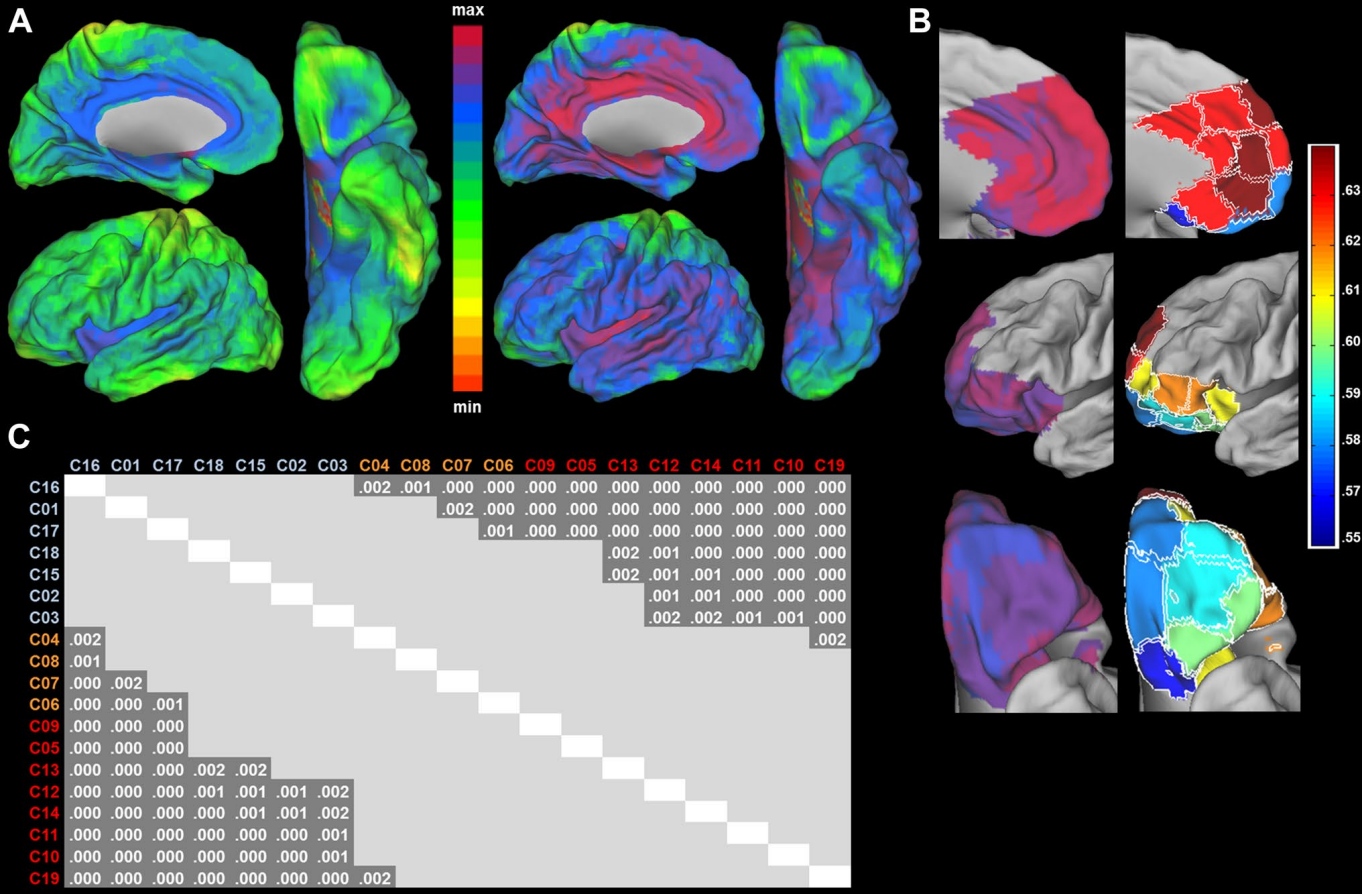
**a** Whole-brain relative signal intensity (*left*) and tSNR (*right*). Values range from 0 to 1.6 for signal intensity and 0 to 24.0 for tSNR. **b** *Left panel* shows the stability of voxel-wise FC profiles (eta^2^) within the OMPFC voxel-wise. Values range from 0 to 0.7. *Right panel* shows the mean stability of voxel-wise FC profiles (eta^2^) for each cluster. **c** Significant pairwise differences of voxel-wise FC profile stability (eta^2^) among clusters (*p* values Bonferroni–Holm corrected)

The orbital part of the PFC is known to be susceptible to BOLD signal loss (Smits et al. 2007). This was also the case in our data set, despite the employment of an imaging sequence optimized for BOLD sensitivity in this area (see “fMRI acquisition” in the Methods section). As shown in Fig. 3a-left, relative signal intensity was lower in subregions of the OFC, but not lower than in many other brain regions outside of OMPFC. The lower signal intensity translated into a lower temporal signal-to-noise ratio (tSNR) in these regions (Fig. 3a-right).

tSNR does not capture the metabolically induced signal fluctuations over time, which are our signal of interest. Therefore, we also computed the stability (or replicability) of a voxels whole-brain FC over acquisitions (first and second resting-state runs) (Fig. 3b). To make a quantified evaluation of regional differences in voxel-wise FC stability, we averaged the stability values per cluster of modules obtained in the parcellation analysis (Fig. 3b-right), and tested for cluster-wise differences. The average eta^2^ ranged from 0.56 in orbital cluster C16 to 0.63 in medial cluster C19. For the clusters occupying the medial wall, the average stability was 0.62, while the average eta^2^ of the orbitofrontal clusters was 0.59. While these minimum–maximum numbers are close to one another, there are nonetheless significant stability differences between orbital and medial clusters, as evidenced by the repeated-measures analysis. Orbital clusters C16, C01, and C17 had the lowest inter-run correlation stability, differing significantly from clusters both at the medial wall (*p* < *0*.001) and the lateral side (*p* < *0*.001). The rest of the orbital clusters (C18, C15, C02, and C03) had a higher eta^2^ which differed significantly only from the medial wall clusters with the higher stability (Fig. 3c). The same analysis with tSNR as a covariate revealed that tSNR had a small, albeit significant (*F* = 30.1, *p* < 0.001), effect on the inter-run FC stability, accounting for only 10% of its variance. Overall, our quality analysis indicates that the magnetic susceptibility artefact, to which EPI imaging sequences are sensitive, does affect signal strength (tSNR) in subparts of the orbital surface compared to other cortical regions, but that its effect on the estimated connectivity profiles of individual voxels is limited.

### Connectivity-based cluster networks in OMPFC

**Fig. 4:**
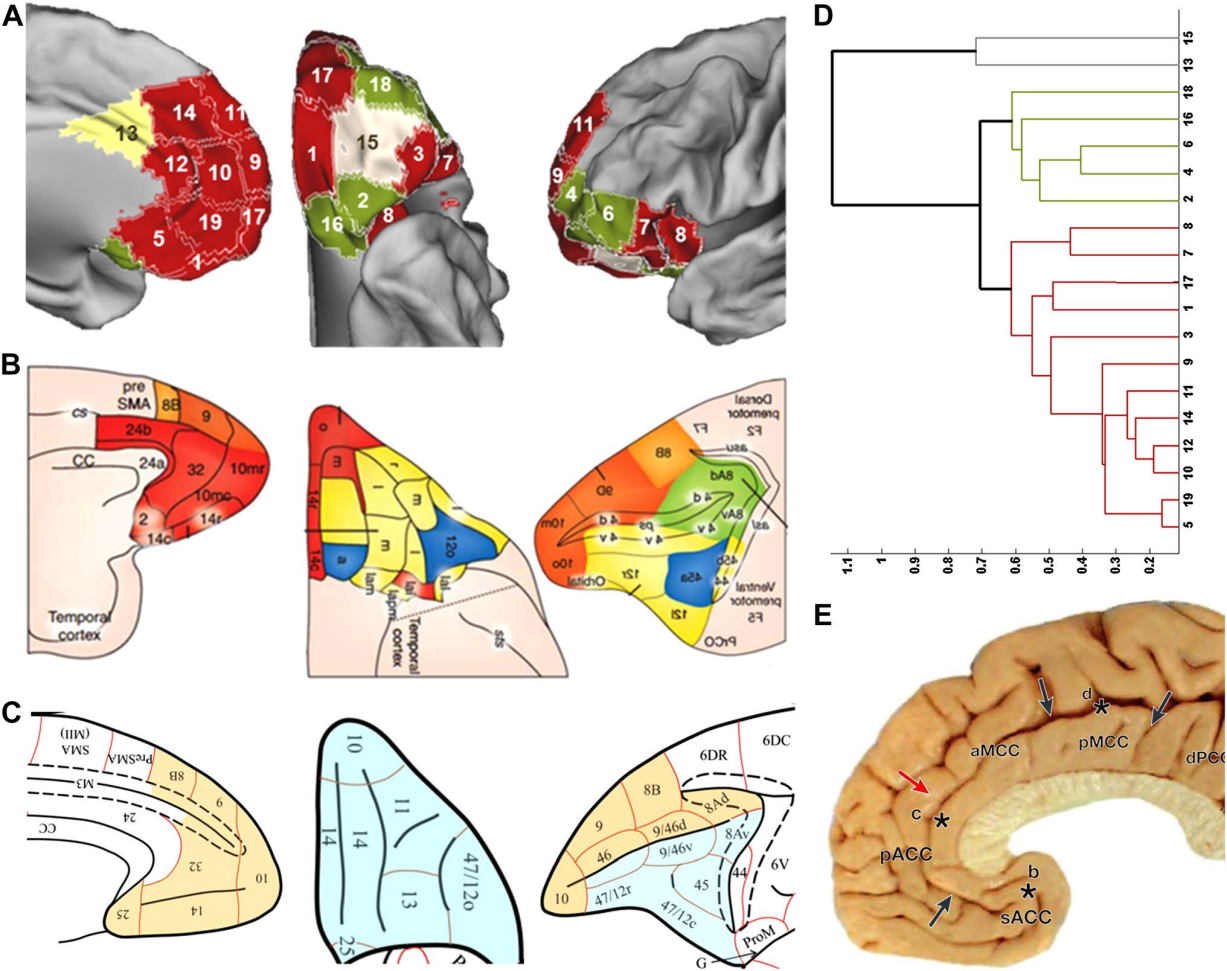
**a** Cortical surface representation of the results of the hierarchical clustering analysis. OMPFC clusters belonging to the medial group are shown in *red*; clusters belonging to the orbital group are shown in *green*; singletons are uniquely *colored*. **b** Medial (*red*) and orbital (*yellow*) networks proposed by Carmichael and Price (1996) and Öngür and Price (2000) (in Price and Drevets 2010). Areas connected to more than one network and believed to act as interfaces for information exchange are seen in *blue*. **c**. Mediodorsal (*orange*) and basoventral (*light blue*) cytoarchitectonic trends described by Barbas and Pandya (1989) (in Yeterian et al. 2012). **d** Dendrogram (average linkage) depicting the similarity of the whole-brain FC profiles of the OMPFC fields within each of the groups. **e** Vogt’s four-region neurobiological model of the cingulate cortex (in Vogt 2005, Box 1). The border between anterior and midcingulate cortex is indicated by the *red arrow*. All pictures reproduced with permission

Overview and discussion of the average cortical and subcortical connectivity profiles of the 19 clusters of modules are presented in the Supplementary Information (see Figure S1 and S2). A possible interpretation of these clusters in terms of cortical fields delineated in cytoarchitectonic studies is documented in table S1. To examine whether the 19 clusters formed extended networks, as proposed by Barbas and Pandya (1989) and Ongür and Price (2000), their averaged connectivity profiles were entered into a hierarchical agglomerative cluster analysis using correlation as the metric of similarity and average linkage (cophenetic correlation coefficient = 0.85; cophenetic coefficient for other linkage methods were as follows: centroid = 0.88, single = 0.84, median = 0.78, complete = 0.77, weighted = 0.77, Ward = 0.75). This resulted in the dendrogram shown in Fig. 4d. At an intra-family distance of 65%, the majority of OMPFC clusters are distinguished in two groups, a “medial” one which includes most clusters on the medial wall and an “orbital” one comprising of orbital and ventrolateral PFC clusters, while clusters C13 and C15 constitute singletons in the solution (see Fig. 4a for a depiction of the spatial distribution of the groups).

The **medial group** was formed by the anterior medial clusters (C05, C19, C10, C12, C11, and C09 linked at an intra-family distance of <0.4) and further included lateral OFC cluster C03, medial OFC clusters C01 and C17 and insulo-opercular clusters C08 and C07. Apart from C13, which is most likely part of the more motor selection-related midcingulate cortex (Vogt 2005, 2016) (see Fig. 4e, and Figure S2 for the unique FC profile of this cluster), all medial wall clusters were grouped together. Finally, in our dendrogram, clusters C07 and C03 were also clustered with the medial group.

The **orbital** group in our dendrogram was formed by the clusters of modules on the posterior and anterior central orbital surfaces (C16, C02, and C18). It also included clusters on the ventrolateral PFC (C04 and C06). Our central OFC cluster C15 was found to have a very distinct whole-brain FC profile and was, thus, not included in the group.

The replicable boundaries described above suggest that the boundaries adhere to but do not systematically coincide with the divisions between networks. The two replicable boundaries flanking the gyrus rectus are located in the transition zone between networks, but do not mark the transition. The boundary located near the convexity between the orbital and medial frontal planes does separate the medial and orbital networks posteriorly (C05 vs. C16) but divides between medial network areas more anteriorly (C05–C19 vs. C01). On the other hand, the boundary lateral to the gyrus rectus/olfactory sulcus posteriorly separates clusters within the orbital network (C16 vs. C02) and more anteriorly marks the outer limit of the medial network (C01 vs. C15, and C17 vs. C18). Similarly, the replicable boundary in the lateral orbital cortex marks transitions between networks (C18 and C02 vs. C03). In contrast, the pregenual boundary (C12-C10 vs. C19) and the more dorsal cingulate sulcus boundary (C14 vs. C13-C12) are between medial network clusters, although the cingulate boundary also marks the posterior limit of the medial network (C13 vs. C12).

## Discussion

Various parts of the human OMPFC play a crucial role in reward, affect, and goal-oriented behaviors and dysfunction in OMPFC fields underlie many severe psychiatric disorders. To improve our understanding of the basic functional organization of the OMPFC, we conducted an in vivo fMRI-based parcellation study of this part of the cerebral cortex. The parcellation was performed at the level of individual participants, by applying graph theory-based analysis techniques to intrinsic functional connectivity measures computed from resting-state fMRI data. Our results show that the functional connectivity between the voxels in the OMPFC has a significant modular structure. The replicability of modules and their boundaries across data sets of the same persons is higher than expected by chance, but lower than for within data set re-analysis. Some of the reliable boundaries co-localized across participants, whereas others are variable across participants. At the higher organizational level, similarities and differences in whole-brain functional connectivity profiles of the clusters of modules pointed towards a segregation of the OMPFC into a medial and an orbital network.

### Consistency with other studies

We found several reliable boundaries that co-localized across parcellations of different participants. To validate this finding, we compare these boundaries with results presented in other studies. More specifically, we asked whether the replicable boundaries observed in this study coincide with boundaries reported in other parcellations schemes.

**Fig. 5:**
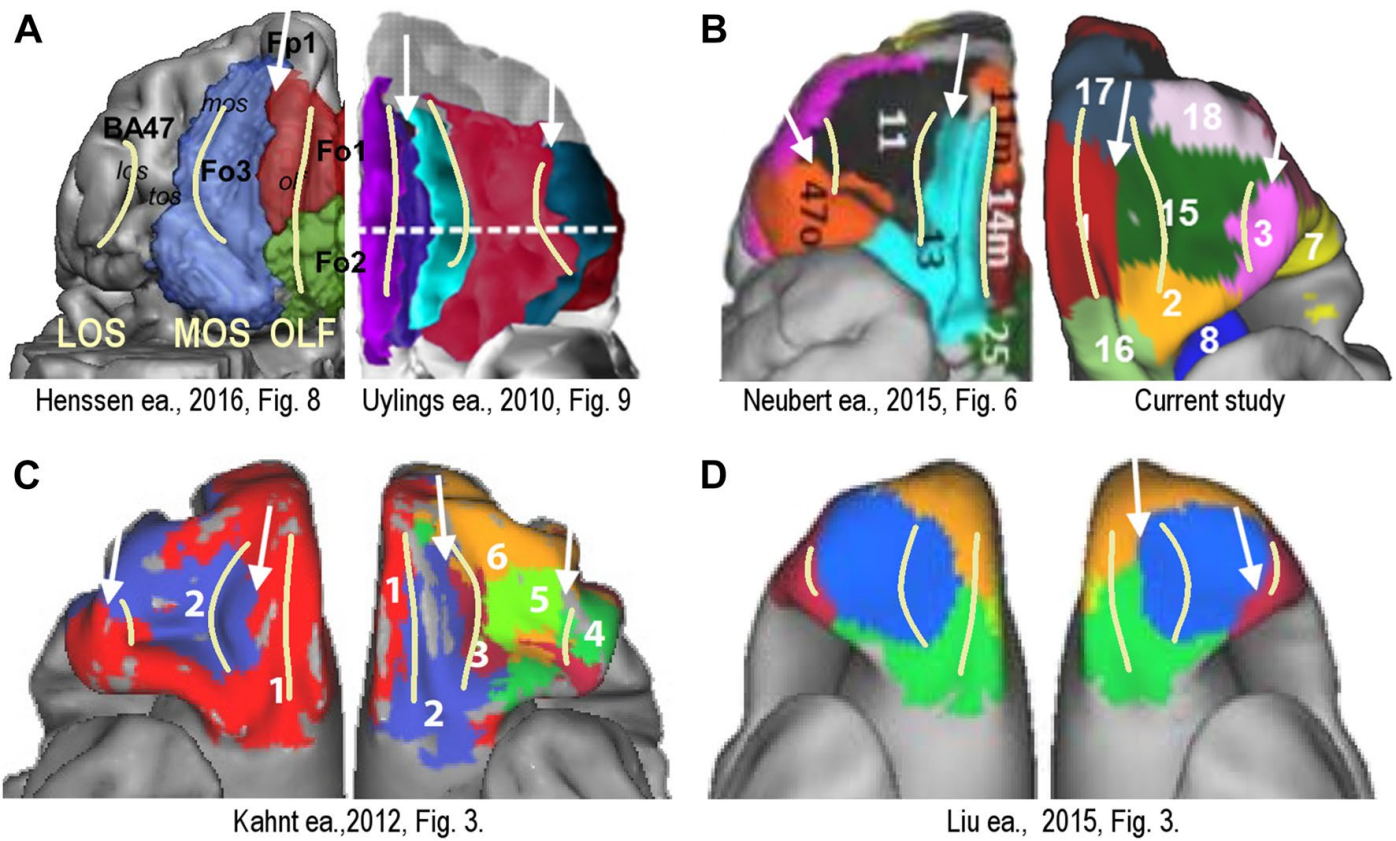
Orbital frontal surface comparison of parcellation schemes from different studies and different modalities with the group results from the current study. *Yellow lines* indicate location of major sulci: *OLF* olfactory sulcus, *MOS* medial orbital sulcus, *LOS* lateral orbital sulcus. *White arrows* indicate boundaries between delineated areas that possibly correspond to the significantly co-localized replicable boundaries found in this study (e.g., Fig. 2a). All pictures reproduced with permission. **a** Cytoarchitectonic parcellations: Henssen et al. (2016) (*left*) and Uylings et al. (2010) (*right*). **b** DWI probabilistic tractography: Neubert et al. (2015) (*left*); resting-state functional connectivity: data from the current study (*right*). **c** Group-wise resting-state functional connectivity from Kahnt et al. (2012): two cluster solution (*left*) and six cluster solution (*right*). **d** Local *grey* matter volume covariation: Liu et al. (2015),* left* and* right* hemisphere

Some recent parcellation schemes for the orbital prefrontal cortex are reproduced in Fig. 5. The parcellations are obtained with different modalities, including cytoarchitectonics [Henssen et al. 2016 (Fig. 5a-left); Uylings et al. 2010 (Fig. 5a-right)], DWI probabilistic tractography [Neubert et al. 2015 (Fig. 5b left)], resting-state functional connectivity [our data (Fig. 5b-right); Kahnt et al. 2012 (Fig. 5c)], and local grey matter volume covariation [Liu et al. 2015 (Fig. 5d)]. Regardless of modality, all these schemes include on the medial side a longitudinal division located between the olfactory sulcus and the medial orbital sulcus, as well as a second longitudinal division on the lateral side, around the position of the lateral orbital sulcus (see white arrows in Fig. 5). The study of Kahnt et al. (2012) shows that these two divisions are also preserved across parcellation scales, as they are present both in the two cluster and the six cluster solution (Fig. 5c, left and right parts, respectively). These boundaries coincide with the location of the orbital replicable boundaries in this study (see Fig. 2a, right most panel).

The different organizational principles along the mediolateral and the rostrocaudal dimension were repeatedly reported in cytoarchitectonic studies of the human and macaque OFC. In the mediolateral direction, cytoarchitectonic boundaries are sharp and well defined (Sarkisov et al. 1955; Ongür et al. 2003; Uylings et al. 2010). Based on a multi-brain histological study, Uylings et al. (2010) proposed a gross subdivision in three partitions from medial to lateral, with the olfactory sulcus and the lateral orbital sulcus grossly marking the boundaries between this three-way division. In contrast, with respect to an anterior-to-posterior subdivision, many authors have noted that there are no sharp boundaries along the anterior–posterior dimension. Rather, they observe a gradual cytoarchitectonic trend that includes a wide transitional zone (Beck 1949; Mackey and Petrides 2010; Uylings et al. 2010). In line with this, individual studies propose a range of further subdivisions along the rostrocaudal dimension. However, these are more variable across schemes. For instance, many schemes posit transverse subdivisions in the medial cortical strip, but the positioning of the division is quite variable. The same is true for the broader midsection of the orbital cortex.

**Fig. 6:**
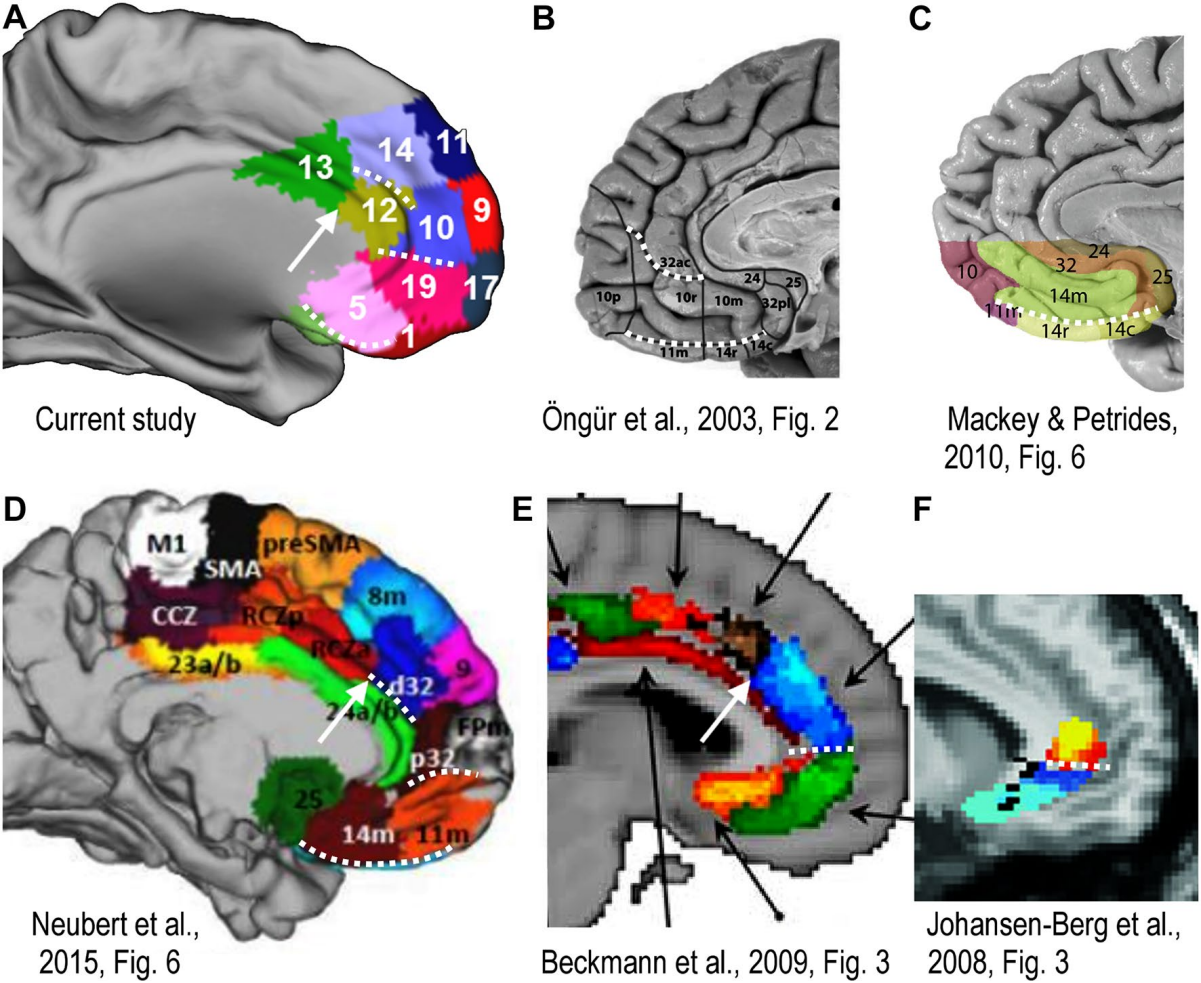
Comparison of parcellation schemes for the medial frontal surface from different studies and different modalities. *Dotted white lines* indicate boundaries between delineated areas that possibly correspond to the significantly co-localized replicable boundaries found in this study (e.g., Fig. 2a). *White arrows* in panels **a, d** and **e** mark potential border between MCC and ACC. *Black arrows* in panel **e** are in the original image and are not interpreted here. All pictures reproduced with permission. **a** Resting-state functional connectivity: data from the current study. **b** Cytoarchitectonic parcellation: Öngür et al. 2003. **c** Cytoarchitectonic parcellation: Mackey and Petrides, 2010. **d** DWI probabilistic tractography: Neubert et al. 2015. **e** DWI probabilistic tractography: Beckmann et al. 2009. **f** DWI probabilistic tractography: Johansen-Berg et al. 2008

On the medial wall, we found replicable boundaries co-localized across participants in three locations: one long stretch around the medial orbital convexity, one anterior to the genu of the corpus callosum, and one dorsally near the (para)cingulate sulcus. When these locations are inspected in other available parcellation schemes of the human medial prefrontal cortex, we find consistent boundaries at these locations across schemes and modalities. An overview of existing parcellations for the anterior medial PFC is provided in Fig. 6. The modalities presented include resting-state functional connectivity [our data (Fig. 6a)], cytoarchitectonics [Öngür et al. 2003; Mackey and Petrides 2010 (Fig. 6b, c)], and DWI probabilistic tractography [Neubert et al. 2015; Beckmann et al. 2009; Johansen-Berg et al. 2008 (Fig. 6d, e, f, respectively)]. The boundaries corresponding to the sites mentioned are indicated with white stippled lined.

Some other boundaries appear to be consistent across parcellation schemes that did not emerge as co-localized replicated boundaries in our analysis, such as the division between the midcingulate and the anterior cingulate cortex (Palomero-Gallagher et al. 2008; Vogt 2005, 2016; see Fig. 4e, red arrow). In our group map, this division emerged as the rostral edge of cluster 13, and similar divisions are observed in other parcellation schemes (see white arrows in Fig. 6a, d, e). This division is known to be associated with a larger functional and connectional discontinuity as it is a watershed region between more task positive and default mode or affective cortical areas (Vogt 2016). In our data, this was confirmed by the fact that in our hierarchical clustering analysis based on functional connectivity profiles of the 19 clusters, cluster 13, which is on the caudal side of that boundary, was placed outside of the two main networks of clusters. The fact that this watershed zone, despite its functional conspicuousness and its presence in several group parcellation maps, did not emerge in our boundary analysis may reflect larger inter-subject variability in its exact location, as it is not specifically linked to a particular anatomical landmark.

### Parcellation replicability

Data on the replicability of parcellation results across data sets have been presented previously, but mostly at the group level and in an informal manner (e.g., Yeo et al. 2011; Sallet et al. 2013). Here, we focused on within participant reliability and used a quantified approach. This allowed us to establish that some aspects of functional organization are robust, and others are not. Replicability at the group level is dependent on reliability at the individual level and inter-subject variability in functional organization. It has been shown that there is quite some variability across subjects in the size and localization of cortical fields and in the location of these fields relative to anatomical landmarks (Uylings et al. 2005; Eickhoff et al. 2005). Of more concern in this study was replicability at the individual level. At this level, organizational features are a likely source of the variability in boundary replicability. First, in cytoarchitectonic studies, some transitions are sharp and clearly delineated, whereas others are gradual and extend over a larger transition zone (Uylings et al. 2010; see “discussion” below). It seems likely that the latter produce less reliable module boundaries also in connectivity-based parcellations. Second, modules are grouped by their interconnection patterns into large-scale functional networks, and it is clear that functional connectivity profiles differ more strongly between modules at the transitions between functional networks than between adjacent modules belonging to the same functional network. It is, therefore, likely that boundaries in the latter case are less reliable. Thirdly, the replicability might also be dependent on the temporal dynamics of couplings between areas, with areas forming functional couplings at shorter time windows being less replicable and areas forming couplings at longer durations showing higher reliability. The temporal dynamics of networks are not yet well understood (Hutchison et al. 2013), but they are likely to play a role when changes in functional organization over time are investigated.

Alternatively, non-functional factors could also play a role. However, in the current study, there does not seem to be an obvious relationship between the reliable boundaries and cortical folding, as some of the co-localized replicable boundaries are associated with a sulcus (e.g., the lateral orbital sulcus, or the (para)cingulate sulcus), and some with the convexity at the boundary between the orbital and medial plane, but others are not (e.g., the pregenual or the medial orbital boundary). In addition, there seems to be no clear relationship between image quality and co-localized replicable boundaries, as such boundaries were found in the orbital plane as well, despite its higher sensitivity to the susceptibility artefact. Moreover, the FC stability was high throughout and differed only slight (albeit significantly) between medial and orbital clusters.

### Network organization

Converging evidence has shown that the orbital and medial PFC are organized in separate networks, e.g., the basoventral and mediodorsal cytoarchitectonic trends described by Barbas and Pandya (1989) and the medial and orbital prefrontal systems of Carmichael and Price (1996) and Ongür and Price (2000) (see Fig. 4a–c). These systems consist of areas that preferentially communicate internally through local cortico-cortical connections. Several studies have demonstrated that the medial and the orbital prefrontal systems are characterized by distinct connections with the rest of the brain (see review in Öngür and Price 2000). In line with this, based on similarities in their whole-brain functional connectivity profiles, the 19 clusters in our study grouped together in two larger networks, one which includes most of the medial clusters and one with clusters from the orbital and ventrolateral cortices.

The medial network comprises the anterior cingulate and medial superior frontal gyrus as well as the adjacent medial edge of the orbital cortex and a small region of the posterolateral orbital cortex. This network is involved in affective and self-centered information processing and has been implicated in mood disorders (Drevets et al. 2008; Price and Drevets 2010, 2012; Rolls 2016). Barbas and Pandya’s mediodorsal trend also includes areas 9 and 10, which have recently been recognized also by others as part of a dorsal prefrontal system (Price and Drevets 2010; Saleem et al. 2014) which is tightly interconnected with the medial prefrontal network of Carmichael and Price (1996) and Ongür and Price (2000). In line with this, our medial clusters C09 and C11, which extend laterally into the dorsal PFC, were also grouped with the medial clusters.

Our clusters C01 and C17, which cover the middle and rostral sections of the medial orbital cortex, were clustered in the medial group. This disagrees with the mediodorsal trend in Barbas and Pandya (1989), which at least in the monkey does not extend into OFC. However, the finding is in accordance with the medial prefrontal network (Carmichael and Price 1996; Ongür and Price 2000), and it does make sense functionally, since the medial orbital cortex has been implicated together with the ventromedial prefrontal cortex in value-based, and thus self-centered, object selection (Rushworth et al. 2007, 2011; Grabenhorst and Rolls 2011). Also implicated in value-based decision-making is the lateral orbital cortex, which has been shown to be activated during learning and updating of object-value associations, both when reward is received (Rushworth et al. 2011) and when expected reward is not obtained (Noonan et al. 2011; Rolls 2016). In line with this role, our lateral orbital cluster C03 was grouped with the medial network, together with the insulo-opercular clusters C08 and C07. Particularly, for the ventral anterior insular cluster C08, this finding confirms the designation of insular subdivision Iai (insula agranular intermediate; Öngür et al. 2003) as part of the medial prefrontal network (Drevets et al. 2008; Saleem et al. 2014), as well as the repeated finding that the anterior insula is functionally linked to ACC (Medford and Critchley 2010; Seeley et al. 2007).

The orbital network (Carmichael and Price 1996; Ongür and Price 2000), together with the ventrolateral prefrontal areas, is primarily involved in the integration of multi-modal sensory stimuli and the coding of the stimuli’s affective value (Price and Drevets 2012). This is reflected in their connections to several sensory as well as striatal areas. The system constitutes a sensory-visceromotor link critical for the guidance of reward-related behavior and the setting of mood (Öngür and Price 2000; Price and Drevets 2012). In Barbas and Pandya’s (1989) basoventral trend, the entire orbitofrontal plane is included, while in Carmichael and Price’s (1996) orbital prefrontal network mainly the cytoarchitectonic subregions of the central orbital surface and parts of the lateral OFC our included. In our orbital group, while posterior OFC clusters C16 and C02 and rostral cluster C18 are grouped together, our central OFC cluster C15 was found to have a very distinct whole-brain FC profile and was, thus, not included in the group. On the other hand, the finding that ventral lateral PFC clusters C04 and C06 are connectionally similar to the rest of the areas in the orbital group agrees with the definition of the basoventral cytoarchitectonic trend which extends on the ventrolateral PFC until the principal sulcus. With regard to Carmichael and Price’s scheme, while the related tracing studies initially included only the orbital surface, a more recent analysis of the connections of the lateral PFC identified a ventrolateral prefrontal system, ventral to the principal sulcus, which is closely connected to the orbital prefrontal network (Price and Drevets 2012).

### Limitations and future perspectives

The in vivo parcellation approach applied here and in other studies of the orbital and/or medial PFC is based on the global patterns of similarity in functional connectivity across voxels (Buckner and Yeo 2014). Because the information involved is very different from cytoarchitectonic features, receptor distributions, and tracer defined connections, no strong claim can be made that the modules delineated correspond to the cortical fields described in invasive neuroanatomical studies. A first caution against over interpreting the modules is the degeneracy inherent in the problem of grouping voxels into modules based on stronger within than between module similarity (Good et al. 2010; Rubinov and Sporns 2011). It has been shown that there are many possible groupings, some with different numbers of modules, that equally well fit the data. While this problem surfaces in stochastic algorithms, such as in this study, it is no less present in studies using deterministic algorithms. This problem needs to be addressed overtly, before the discussion of correspondence between modules and cortical fields becomes meaningful. It implies that additional information needs to be considered when interpretation the results. One example of this additional information is replicability across data sets, as was used in this study. Another example is consistency across modalities, as was recently explored in a large study from the Human Connectome Project (Glasser et al. 2016).

A second point of caution in equating modules with established cortical fields is that the critical amount of similarity for grouping voxels into modules is indirectly dependent on parameters of the algorithms, such as the number of centroids in clustering approaches, the number of components in linear decomposition methods, and the threshold used to binarize the correlation matrix in our approach. While this does not necessarily affect the quality and reliability of the parcellation results, it does pose a problem for interpreting and comparing results across studies. Cytoarchitectonic cortical fields may not coincide with functionally relevant subdivisions. For instance, a number of functionally meaningful subdivisions of BA6 have been proposed (Barbas and Pandya 1987; Matelli and Luppino 2001; Petrides and Pandya 2006). In addition, different functionally meaningful cortical divisions can emerge from the same FC data by focusing the analysis on local gradients rather than global FC profile similarity (Buckner and Yeo 2014). In addition, parameter settings resulting in fewer but larger modules might group cortical fields or subfields, whereas settings yielding smaller modules might divide fields into functional subunits. Moreover, optimal parameter settings might differ for different individual data sets and even different regions within one data set. This scaling problem, which is not yet well understood, limits the comparison of results from different studies. At the same time, it is promising that several regional segregation features emerge in a range of studies in spite of differences in modality or analysis tools.

Another limitation with respect to the present results is that we were not able to make a clear differentiation between reliability at the individual level and replicability at the group level. While we had solid statistical support for delineating co-localization of boundaries in certain voxels at the group level, we are not able to establish whether the lack of co-localization in other voxels was due to no boundary being present or boundaries being present but with too much inter-individual variability for co-localization to reach significance. This is an important difference when it comes to evaluating the reliability of parcelletion methods, but also in the study of cortical functional organization at different resolutions (the scaling problem discussed above), in different modalities and in different populations. While the two available data sets per participant allowed us to establish that there was a significant amount of replication in the boundaries found, it did not provide statistical power to establish the significance of the replicated borders at the individual level. As a consequence, we could not study inter-individual variability in the location of these borders. Thus, while we did not find the expected consistent boundary between mid and anterior cingulate cortex, we were not able to disentangle whether this is because it is not sharply defined (i.e., a transition zone), or that it is a clear connectional discontinuity but with larger inter-individual variability in its location. Having a statistical quantification of borders based on larger number of resting-state measurements per individual would allow us to resolve this ambiguity. It would provide the opportunity to delineate first-level reliable organizational features, which can then be studied at the second or group level across participants. Such an approach would make it possible to address quantitatively what the gain is of having more and longer resting-state runs per individual and what would be the optimal amount of data. It would also allow quantifying to what extent for instance resting-state functional fMRI and diffusion weighted imaging lead to replicable features, or whether each method exhibits modality specific replicable features and, therefore, highlights each unique aspects of the cortical functional organization.

A third point of caution in the interpretation of our results is related to acquisition quality differences between the orbital and medial prefrontal cortex. While our quality metrics and results confirm that parcellation at the OFC is feasible, MR signal quality, and connectivity stability over runs was somewhat lower in the medial and rostral OFC. This may affect somewhat the quality of the FC maps for these regions. In the future, studies that take advantage of advanced MRI hardware options might further minimize the signal quality loss and yield more complete descriptions of the FC in these brain regions.

## Electronic supplementary material

Below is the link to the electronic supplementary material.

Supplementary material 1 (DOC 8016 KB)
