# Supplementary material for: Human orbital and anterior medial prefrontal cortex: Intrinsic connectivity parcellation and functional organization
Source: Brain Struct Funct. 2017 Mar 2;222(7):2941–60. doi: 10.1007/s00429-017-1378-2 (PMC5581738; doi:10.1007/s00429-017-1378-2)

**Running Title:** Human orbitomedial PFC: connectivity parcellation

**Supplementary Information**

**Methods**

1. Group-wise clustering: weighted merging of parcellation pairs.

Merging parcellations at the end of each iteration was accomplished by averaging the COMs of assigned modules in a weighted manner. Prior to the first iteration, each module of each parcellation received an weight of 1.0. Whenever during the merging process a module remained un-assigned due to different numbers of modules in the two parcellations, that module was penalized by halving its weight – since the least fitting module(s) remain unassigned. Whenever a parcellation remained unassigned, due to an uneven number of parcellations, all its modules were penalized (again because the unassigned one is the least matching one) by an amount that was proportional to the progress of integration. The weighting was proportional (i.e., 0.5 plus the inverse of the number of parcellations remaining at this iteration) because the farther into the integration process the larger the impact of the merger on the final result. As a consequence of both weighting rules, the least fitting modules' influence during averaging with another module at the next level was reduced. The final set of COMs, i.e., after the last iteration when only one merged parcellation remained, was “cleaned” in two ways: (1) modules with a weight < 0.5 were eliminated from each cluster; (2) the 10% modules farthest away from the final group COM were eliminated from each cluster. Finally, for each cluster a new COM was computed as the average coordinates of all voxels with a positive within-module degree z-score (Guimerá and Amaral, 2005) (i.e. only voxels/nodes tightly connected within their module had an influence on the clusters’ COMs).

2. Replicability of intrinsic FC parcellation: creation of random module models.

The null hypothesis for this analysis was that the spatial location and boundaries of the observed modules are random, i.e., not dependent on information in the connectivity matrix. Null modules based on randomizing connectivity weights or edges in the connectivity matrix result in spatially highly fragmented and discontinuous modules, and therefore cannot capture the required limit of random spatial replicability of modules. Therefore, we created null models with a similar number of modules as in the observed parcellation randomly positioned within the voxel space of that parcellation. First, for each module an anchor point was randomly positioned with the constraint that it must be a critical distance away from each of the already positioned anchor points for other modules. The critical distance was the smallest distance between any two modules’ center of mass in the observed parcellation. Second, each voxel in the parcellation space was assigned to the nearest module anchor point, and the set of all voxels assigned to an anchor point constituted a randomly located module. A separate random model was created for the parcellation of the first and second resting state data set of each participant and the quantified comparisons between the random models of each participant were used as a control condition against which the same quantifications for the observed solutions were compared.

**Results: *Whole brain FC profiles of OMPFC subdivisions***

In order to validate our OMPFC partition we compared the relative position and extent and the FC profiles of our 19 clusters, shown in Figures S1 and S2, with various cytoarchitectonic maps (see Figure 5 and 6 in the paper) and macaque anatomical connectivity fingerprints.

The functional connectivity maps presented in Figures S1 and S2 were created from the modules-specific Fisher-Z transformed Pearson correlation maps that were created per participant (see paper, “Materials & Methods”, subsection “Similarity of whole-brain FC profiles of the OMPFC subdivisions”). For every cluster of modules, a separate voxel-wise one-sample *t*-tests was performed against the null hypothesis of absence of connectivity between the clusters and the rest of the brain. Multiple comparisons were cluster-level corrected (Chumbley and Friston, 2009) and the cluster extent necessary for the correction was determined with Monte Carlo simulations (implemented in Brain Voyager QX 2.8; Goebel et al., 2006). The voxel-level threshold of each map was set at *p* = .01, uncorrected, and the functional image matrix (79 X 95 X 69 voxels) was modeled in an iterative procedure using the estimate of the map’s spatial smoothness. Following 10.000 simulations, the minimum cluster-level threshold that yielded *p* = .005 was selected. We subsequently used CARET (http://brainvis.wustl.edu/wiki/index.php/Caret:About) to project the cluster-level thresholded FC maps of each cluster on a 3D cortical surface.

While a cortical surface based visualization of results provides an integrated overview of the spatial distribution of functional coupling, it does not allow visualization of subcortical FC. Because of the instrumental role OMPFC in emotional processing, mood setting and reward representation (Drevets et al., 2008), we chose to provide a separately visualization of the same analysis results focusing on the subcortical aspect of the FC profiles described above by means of fingerprint plots. To this end ROI masks were created for a range of subcortical structures. For the amygdala, hippocampus, thalamus, caudate and putamen we created bilateral masks using the corresponding AAL labels. Masks for the ventral striatum and hypothalamus were created using the definition and instructions of Tziortzi et al (2011). The ventral striatum mask covers the area functionally defined as ventral striatum in connectivity studies and nucleus accumbens, ventral medial caudate nucleus and rostroventral putamen (Tziortzi et al., 2011). For the thalamic mediodorsal nucleus and pulvinar, we placed spherical ROIs of 8mm radius at MNI coordinates x=-6, y=-18, z=8 and x=-15, y=-33, z=6 respectively (Zou et al., 2009). The mask for the periaqueductal gray was manually drawn in MRIcron (Rorden and Brett, 2000).

The subcortical ROI masks were used, in combination with the cluster-level thresholded t maps, to extract the strength of the significant positive connections in each subcortical area. For each cluster, a vector was created that reflected the average Fisher Z transformed connectivity strength (Taylor et al., 2009) between the specific cluster and the significant voxels in each subcortical ROI. The average strength of each subcortical ROI with each cluster was normalized by dividing all values by the maximum average strength of each cluster. The resulting vectors were represented in fingerprint plots that visualize the subcortical FC of each cluster on a zero to one scale.

***1. Orbital Clusters***

On the orbital surface of the PFC we observed 7 clusters of modules: C01, C02, C03, C15, C16, C17 and C18. The cortical and subcortical FC profiles of each of these orbital cluster is visualized in Figure S1 and Table 2 in the paper gives an overview of all clusters’ candidate anatomical substrates. On the medial side we found the two clusters C01 and C16 demarcating a longitudinal strip parallel to the olfactory sulcus, while on the lateral side cluster C3 covered the orbital cortex lateral to the lateral orbital sulcus. This demarcation of a lateral and medial zone seems to coincide with the consistent boundaries found across data sets from same and different individuals (see paper, Figure 2A, left). The midzone between these boundaries comprised of cluster C15 and C2.

In cytoarchitectonic studies an anterior-posterior and a medial-lateral trend have been observed such that laminar differentiation of (mainly) layer IV and anatomical connectivity with distributed cortical and subcortical fields varies along these axes (Uylings et al., 2010; Cavada et al., 2000). These anterior-posterior and medial-lateral cytoarchitectonic and connectional trends are reflected in functional imaging studies (Kringelbach and Rolls, 2004; Kringelbach, 2005). In our results, along the anterior-posterior dimension, clusters C01, C15 and C03 are anterior, whereas C16 and C02 are posterior. In the medial-lateral dimension, clusters C01, C15 and C03 spatially agree with the recent subdivision proposed by Uylings et al. (2010) into an area 11, and areas 47m and 47l, with the olfactory sulcus and the lateral orbital sulcus grossly marking the boundaries between this three-way division. In the anterior-posterior direction, the spatial location of our division line is located somewhat more posterior than the division proposed by Uylings et al. (2010), and agrees more with a transition zone noted by Beck (1949). Many authors have noted, however, that the anterior-posterior division is not marked by sharp boundaries but rather constitutes a gradual cytoarchitectonic trend that includes a quite wide transitional zone (Uylings et al., 2010) as opposed to the medial-lateral division which is marked by sharper transitions. The FC profiles of our clusters vary along these dimensions, in agreement with macaque tracing studies (Cavada et al., 2000; Ongür and Price, 2000), as detailed below.

Cluster C01.

On the medial edge of the orbital side cluster C01 occupies all but the caudal part of the gyrus rectus and extends to the medial orbital gyrus. The COM of this cluster is located in the medial olfactory sulcus (-8, 38, -25). Cluster C01 has - in addition to coupling with the immediately adjacent ventral medial regions - confined connectivity with the posterior cingulate region (area 23d of Vogt et al., 2005) and the visual part of the precuneus (Margulies et al., 2009), and with the dorsal medial cortex (presumably BA 9), which was recently implicated in social cognition (Rushworth et al., 2013). Ventrally this cluster has focal coupling with the later orbital gyrus (our cluster C03). On the lateral side C01 is functionally connected with the ventral prefrontal cortex extending towards the anterior bank of the precentral sulcus, the angular gyrus and the lower aspect of the lateral temporal lobe. C01’s pattern of FC largely overlaps with the anatomical connections seen in the presumed homologue macaque area 14 (reviewed in Yeterian et al., 2012; Morecraft et al., 1992; Carmichael and Price, 1996; Petrides and Pandya, 2012). The medial OFC is implicated in the value-weighting of choice options and the value-representation of chosen options (Rolls and Grabenhorst, 2008; Rushworth et al., 2011).

Cluster C15.

The cluster just lateral to C01 is cluster C15. It covers the anterior and part of the posterior orbital gyrus and laterally extends towards the medial orbital gyrus, with its COM (-27, 39, -18) at the anterior orbital gyrus. In terms of functional coupling, C15 is in many respects the opposite of cluster C01. On the medial side it is connected with the dorsal aspect of the precuneus instead of the ventral limbic portion (Margulies et al., 2009), and with mid-cingulate motor areas (Amiez and Petrides, 2014) instead of the anterior cingulate gyrus. On the lateral side, C15’s signal is significantly coupled with the anterior aspect of the supramarginal and the intraparietal instead of the angular gyrus, with the middle (presumed area 46; Goulas et al., 2012) instead of the inferior frontal gyrus, and with the posterior instead of the midsection of the lateral temporal lobe. Moreover, there is also coupling with the frontal eye fields and the dorsal-anterior (cognitive) subdivision of the insula (Kurth et al., 2010). This distributed pattern resembles frontoparietal and ventral attention network connectivity (Yeo et al., 2011) and has been associated with attention engaging processes during task execution (Duncan and Owen, 2000; Fox et al., 2006; Corbetta et al., 2008; Stiers et al., 2010). A spatially co-localized albeit smaller cluster identified in a previous parcellation study showed a similar pattern of FC with task-associated brain regions (cluster 3 in k=6 solution of Kahnt et al., 2012). C15’s profile of FC overlaps with the connectivity profile ascribed by tracing studies to macaque area 11 (Yeterian et al., 2012).

Cluster C03.

Our most lateral cluster on the orbital plane is cluster C03, covering the lateral orbital gyrus with its center at the mediocaudal part of the gyrus (COM -36, 34, -16). This spatial location corresponds to areas 47/12 (Petrides and Pandya, 1994; Mackey and Petrides, 2009; Ongür et al., 2003) and 47l^2^ (Uylings et al., 2010). The FC of C03 differs from that of C15 and is again more similar to that of C01, but shows also distinctive features that parallel what is known for the connectivity of area 47/12 in the macaque (Yeterian et al., 2012). C03 has no posterior coupling in the medial wall, whereas its cingulate gyrus coupling is in between the subgenual focus of C01 and the mid-cingulate region of C15, with a strong focus in the anterior subpart of the mid-cingulate gyrus. On the lateral side, C03 shares with C01 the FC with the ventral aspect of PFC - albeit with a strong focus somewhat lower on the operculum - up to the posterior aspect of the middle frontal gyrus. However, C03 shows coupling with the dorsal aspect of the lateral PFC, in contrast to C01 which lacks such connectivity. Further, we observe again that medial cluster C01 and lateral cluster C03 are functionally coupled. This, together with C03’s strong coupling with all three major input nuclei of the basal ganglia, and with the amygdala and hippocampus, is consistent with the presumed role of the lateral OFC in learning and updating of the reinforcer and value association of choice options (Rushworth et al., 2011), information assumed to be communicated to the medial OFC (our C01) during decision making.

Cluster C16.

In the posterior OFC our medial-posterior cluster C16 occupies the caudal part of the gyrus rectus (COM -15, 15, -19) and is the spatial homologue of subregion 14c (Ongür et al., 2003; Mackey and Petrides, 2009). The FC pattern of C16 is similar to that of C01 on the posterior and ventral medial wall, but differs with respect to its dorsal anterior connectivity since it is functionally coupled with the dorsal cingulate cortex rather than the dorsal medial superior frontal gyrus as C01. Additionally, C16 shows FC with the anterior middle frontal gyrus rather than the operculum, parietal coupling extending in the IPS, inferior temporal rather than middle temporal connectivity and FC with the ventral anterior insula and the central orbital cortex (our C15).

Cluster C02.

Our lateral posterior cluster C02 in the caudal OFC covers the extent of the posterior orbital gyrus (COM -26, 24, -21). The FC profile of cluster C02 resembles that of lateral orbital cluster C03, but overall it is less strong. There are noteworthy differences, however, which agree with the tracer connectivity reported for macaque area 13 (Yeterian et al., 2012; Petrides and Pandya, 2012). While C02's anterior medial connectivity is similar to that of C03, including the rostral mid-cingulate focus, C02 also shows posterior cingulate gyrus coupling. Moreover, C02’s signal is significantly coupled with both the inferior temporal cortex of the ventral visual stream and lower visual cortical areas, (i.e. fovea representation). Conversely, C02 lacks the strong posterior lateral PFC connectivity that characterized C03.

Cluster C17.

The anterior boundary between clusters C01-C15 and C17-C18 in our parcellation coincides with the boundary proposed by Uylings et al. (2010) for the division between the orbital cortex and the frontopolar cortex, comprising Brodmann's area 10 (Petrides and Pandya, 1994; Ongür et al., 2003; Mackey and Petrides, 2009). Human fMRI studies have implicated parts of BA10, in addition to lateral and medial orbital cortex, in reward-oriented decision making (Rolls and Grabenhorst, 2008). In line with this, the FC profiles of the fully differentiated cortical fields anterior to OFC appear as gradual extensions of the patterns observed in the OFC. Our medial cluster C17, located in the ventromedial part of the frontopolar surface and centered at the frontomarginal gyrus (-13, 61, -14), shares many connectivity characteristics with medial orbital cluster C01. The functional coupling pattern of C17 differs from that of C01 only in a few specific respects. On the orbital side C17 is functionally connected to the medial and not to the lateral side. In the lateral PFC, C17 showed no evidence of connectivity with the posterior operculum, but it does have functional coupling with the superior frontal gyrus (BA 9, our cluster C11). Lastly, C17 was the only cluster on the orbital plane for which we found temporal pole coupling. This confirms the reported DWI connectivity between an orbital division of area 10 and the temporal pole in humans (Liu et al., 2013). The temporal pole is considered paralimbic cortex and believed to be functionally implicated in the integration of emotional responses with highly processed sensory information and to be instrumental in the evaluation of emotional states (van Eijndhoven et al., 2013).

Cluster C18.

Adjacent to C17 in the anterior OFC lies cluster C18, immediately rostral to orbital cluster C15, on the lateral part of the frontopolar cortex, a cluster centered at the anterior orbital gyrus (COM -30, 54, -13). Cytoarchitectonic parcellations do not agree on whether the cortical surface of the ventral frontal pole consists of one extended region or more divisions (Petrides and Pandya, 1994; Ongür et al., 2003; Drevets et al., 2008; Carmichael and Price, 1994; Hof et al., 1995). A recent cytoarchitectonic map showed that the human frontopolar cortex consists of one lateral frontopolar and one medial frontopolar division which are implicated in cognition, working memory and perception, and affective and social processing respectively (Bludau et al., 2014), while DWI imaging studies in humans (Catani et al., 2012; Liu et al., 2013; Moayedi et al., 2014; Neubert et al., 2014; but see Sallet et al., 2013 where one single presumed BA10 was identified) have also delineated more than one regions in the frontopolar cortex. Both our C17 and C18 are part of the lateral frontopolar subdivision Fp1 of Bludau et al. (2014) parcellation. Noteworthy is that in the only other FC parcellation of the OFC (Kahnt et al., 2012) the frontopolar-orbital surface of the left hemisphere was consistently divided in at least two clusters, across the range of k=2 to k=7 clustering solutions. In addition, a meta-analysis of PET and fMRI data has suggested functional subdivisions in frontopolar PFC (Gilbert et al., 2006). Although, statistical significance of FC for C18 was weak (mainly due to low inter-subject consistency), the features that could be discerned suggested that C18 is most likely a continuation of orbital cluster C15. C18 and C15 are the only clusters in this part of our parcellation patch that have clear functional coupling with the intraparietal sulcus, associated with the dorsal attention system (Fox et al., 2006; Corbetta et al., 2008). Additionally, they both have significant coupling with the posterior part of the lateral temporal lobe.

Subcortical connectivity of orbital clusters.

Orbital posterior clusters C16 and C02 together with the lateral C03 constitute the link of the OFC with subcortical structures in our parcellation. All three clusters showed strong connectivity with the striatum. While for the medial cluster C16 and central C02 this was confined to ventral striatum and caudate nucleus, C03 showed equally strong coupling also with the motor-related putamen, consistent with its strong affinity with the lateral PFC and the precentral gyrus. This pattern was also observed by Kahnt et al. (2012) who found that the medial part of posterior OFC had more FC with limbic striatum while the lateral part projected more to dorsolateral striatal area. Anatomical connections with the ventral striatum have been found in tracer studies for posterior regions of the macaque orbital cortex (Haber et al., 1995; Ferry et al., 2000), whereas the caudate nucleus has been found to receive projections from the lateral OFC (Selemon and Goldman-Rakic, 1985; Garrett et al., 1986; Haber et al., 1995). Putamen has been functionally linked with the lateral OFC, since inactivation of putamen in the macaque leads to selective impairment of reward history-based action selection (Muranishi et al., 2011). Moreover, individual variation in the monamine levels of monkey caudolateral OFC and the putamen interact to predict reversal learning performance (Groman et al., 2013). Functional coupling with the hippocampus was observed for our lateral cluster C03. FC of the lateral OFC with the hippocampus has been associated with learning of social cues (Ross et al., 2013), and is consistent with the role of lateral OFC in learning and updating stimulus-reward associations (Rushworth et al., 2011). In line with evidence in the macaque (Carmichael and Price, 1996; Amaral and Price, 1984; Ghashghaei et al., 2007) we found amygdala-OFC coupling for our posterior-lateral cluster C03. Communication between the amygdala and the lateral OFC appears to be essential for updating of stimulus-reinforcer associations (Baxter et al., 2000; Rudebeck and Murray, 2011; Rushworth et al., 2011) and to be involved particularly in the processing of biologically salient stimuli such as food (Morris and Dolan, 2001). It should be noted here that tracing studies in macaques have found dense and highly efficient projections from the amygdala to posterior OFC (our cluster C02) that are believed to be critical for processing emotional content (Ghashghaei et al., 2007; Timbie and Barbas, 2014). However, we observed significant FC with the amygdala only for our lateral C03 and not the posterior OFC C02.

***2. Lateral Clusters***

Cluster C06.

On the lateral boundary of our parcellation region several clusters of modules were found. Directly lateral of our lateral OFC cluster C03, we found cluster C06. C06 occupies the most anterior tip of the middle frontal gyrus (COM -45, 46, -4). C06’s FC pattern resembles that of cluster C03. Similar to C03, C06 shows medial coupling with the ventral PFC and anterior paracingulate gyrus, and lateral coupling with dorsal and ventral lateral PFC. Conversely, unlike C03, C06 shares C18’s coupling with the intraparietal sulcus and the middle inferior temporal lobe. Finally this cluster has widespread connections throughout the entire orbitofrontal plane. The extension of the FC pattern from lateral and anterior orbital surface on the ventrolateral PFC is consistent with the interpretation by Uylings et al. (2010) that the lateral OFC boundary should be drawn more laterally than most studies have recognized. Subcortically, C06 shares with C03 the significant coupling with the dorsolateral and ventral striatum, but communicates broadly with the thalamus instead of the hippocampus and amygdala.

Cluster C04.

Anterior to C06 and dorsal to C18, at the junction of the superior and middle frontal gyrus (COM -27, 60, -2) we delineated cluster C04. This cluster shares many FC features with C06, but lacks its coupling with the OFC. Medially it shares C06's medial dorsal connectivity, but lacks its ventral coupling and has a stronger focus on the cingulate gyrus. Additionally, C04 shows posterior cingulate and precuneus connectivity, features which are absent from C06’s map. On the lateral side, C04 is, similarly to C06, connected with the dorsal and ventral PFC, the middle inferior temporal gyrus and the intraparietal sulcus and the supramarginal gyrus. Finally, the subcortical connectivity of C04 parallels that of C06, although with a more specific link to the mediodorsal thalamic nucleus.

Cluster C07.

Posterior to C06, on the ventral part of the frontal operculum, our parcellation includes cluster C07, with its COM (-46, 29, -5) at the pars triangularis of the inferior frontal gyrus. C07 overlaps spatially with the ventral part of area 45A (Petrides and Pandya, 1994). Although our C07 includes only part of the cortex designated as area 45A, the pattern of its FC largely overlaps with the profile of area 45 seen in the macaque (Yeterian et al., 2012; Petrides and Pandya, 2012) and greatly resembles clusters delineated in previous human resting-state parcellations (Kelly et al., 2010; Ford et al., 2010; Goulas et al., 2012; Neubert et al., 2014; Margulies and Petrides, 2013). C07 is functionally connected to the dorsal and ventral medial PFC, to the posterior cingulate extending on the precuneus, has limited coupling with mainly the caudolateral OFC, and on the lateral side is functionally connected with the dorsal superior frontal gyrus, the temporal-parietal junction and the superior and middle temporal lobe. Subcortically C07 is strongly coupled with the amygdala, the hippocampus, the thalamus, the mediodorsal thalamus, the hypothalamus, the caudate, the putamen, and somewhat less strongly with the ventral striatum.

Cluster C08.

Moving into the lateral sulcus, our parcellation yielded cluster C08, which spans the anterior part of the insular cortex and is centered at the lateral fissure (-35, 20, -9). Aside from strong intra-insular connections across the entire extent of the structure, C08 exhibited significant widespread connectivity with the dorsal medial cingulate and paracingulate cortex. Moreover, there is significant coupling with the entire extent of the superior and the middle temporal cortex and the temporal pole, the parietal and temporal opercula, the frontal operculum and the inferior frontal gyrus. This FC profile is consistent with the anatomical connections of the primate anterior insula (Augustine, 1996; Flynn et al., 1999; Mesulam and Mufson, 1982; Vogt and Pandya, 1987; Mufson and Mesulam, 1982). Similar connectivity has also been found in human resting-state connectivity and DWI studies (Cauda et al., 2011; Nelson et al., 2010; Deen et al., 2011; Cerliani et al., 2012). At the subcortical level, C08 is strongly coupled with the amygdala, hippocampus, thalamus, hypothalamus, ventral striatum, caudate and putamen, and somewhat less strongly with the mediodorsal thalamus. In the macaque, connections of the insula have been observed with these structures (Flynn et al., 1999; Augustine, 1996; Gallay et al., 2012; Chikama et al., 1997). Connections with the amygdala and the hippocampus have been confirmed also for humans in a recent DWI study (Cerliani et al., 2012). The anterior insular cortex links stimuli with emotional valence via intense, reciprocal amygdala connections and generally serves as a limbic integrative center for autonomic and sensory function (Devinsky and D’Esposito, 2004). The anterior insula/frontal operculum together with the dorsal ACC are considered core regions of a “salience network” responsive to stimuli relevant to emotional and/or motivational states and functionally linked with subcortical structures such as the extended amygdala, the mediodorsal thalamus and the hypothalamus, which are also involved in such responses (Medford and Critchley, 2010; Seeley et al., 2007).

***3. Medial Clusters***

On the medial wall of the PFC our parcellation yielded eight clusters of modules (see Figure S2 for the cortical and subcortical FC profiles of each medial cluster and Table S1 for an overview of the clusters’ candidate anatomical substrates). Their overall spatial layout is in agreement with the organization described in the four-region neurobiological model of the cingulate cortex (Vogt et al., 2005; see paper Figure 4E). According to this model, the prefrontal part of the cingulate gyrus is divided in a perigenual part, the anterior cingulate cortex (ACC), which includes cortical areas 25, and 24 and 32 proper, and a middle cingulate cortex (MCC) region comprising cortical areas 24' and 32' (Palomero-Gallagher et al., 2008). However, even within the ACC, rostrocaudal variation in cytoarchitectonic features is observed in both areas 24 and 32 (i.e., the cingulate and paracingulate gyri respectively; Koski and Paus, 2000; Yu et al., 2011; Margulies et al., 2007). The subdivisions of ACC's areas 24 and 32 are accompanied by functional differentiation (Vogt, 2005; Vogt et al., 2013; Palomero – Gallagher et al., 2013) a well-documented finding in human MRI studies (Yu et al., 2011).

In line with the above, in our parcellation we observed a chain of four clusters situated rostrocaudally around the corpus callosum (clusters C05, C19, C12 and C13). In the perigenual medial wall we found no segregation into more ventral and more dorsal clusters. Instead, our clusters C05 and C19 spatially covered the region of both area 24 and 32, and beyond. More posteriorly, this division was apparent, with clusters C12 and C13 covering mostly the cingulate gyrus, as does area 24/24' and our clusters C10 and C14 extending on the paracingulate cortex, similarly to area 32/32'. On the cingulate gyrus the boundary between the most posterior cluster C13 and the more anterior clusters C12 and C14 spatially corresponds to the distinction between the ACC and MCC regions of Vogt’s model. More importantly, this discontinuity is also reflected in the FC profiles of these clusters. Although our ACC clusters constitute discrete subdivisions, they show rather gradual transitions in their cortical FC patterns (as opposed to the abrupt changes we observed at the orbital plane), with all clusters sharing a clear Default Mode Network (DMN) signature (Buckner et al., 2008). On the other hand, the pattern of subcortical connectivity that we observed for these clusters is more variable and distinct, with clusters having an overlapping yet distinct subcortical signature. For instance, a signature can be deciphered when taking into account the connectivity with the amygdala. Only C19 and C05 exhibit pronounced amygdala connectivity while the rest show modest levels of connectivity, as predicted by the amygdala- medial PFC connectivity of the macaque (Ghashghaei et al., 2007).

Cluster C05.

The most ventral cluster within ACC is cluster C05. It occupies the subgenual cingulate cortex and extends anteriorly and ventrally beyond the cingulate sulcus. It is centered (-3, 29, -14) at the paralimbic tier of the medial wall adjacent to the anterior subcallosal (paraolfactory) sulcus. Its spatial location and extent covers largely to cytoarchitectonic areas 25 and s32 (Petrides and Pandya, 1994; Palomero – Gallagher et al., 2013). On the medial side C05 is functionally connected to the posterior cingulate gyrus and the precuneus, the perigenual cingulate cortex and the medial superior frontal gyrus. On the lateral side C05 is significantly coupled with the superior and inferior frontal gyrus, the ventral anterior insula and temporal pole and the anterior-medial and lateral temporal lobe. Finally, on the orbital surface, C05 shows FC with the medial and lateral orbital cortex (our C01/C16 and C03). This FC profile corresponds well to the connectivity profiles ascribed to macaque areas 25 and 32 (Yeterian et al., 2012; Vogt and Pandya, 1987). Further, C05 shows connectivity with the angular gyrus extending towards the temporoparietal junction. This connectivity pattern is in keeping with human resting-state FC and DWI connectivity studies (Margulies et al., 2007; Yu et al., 2011; Johansen-Berg et al., 2008; Beckmann et al., 2009).

Cluster C10, C14, and C19.

Dorsal to C05, along the paracingulate gyrus, we have found clusters C19, C10, and C14 which spatially cover the subdivisions of area 32 (C19 and C10 to p32 and C14 to d32; Palomero-Gallagher et al., 2013). C19’s COM (-4, 45, -11) is situated at the cortex above the 1st supraorbital sulcus, anterior of the corpus callosum’s genu, and covers both cingulate cortex and beyond. C10’s COM (-5, 51, 2) is located at the dorsal pregenual cortex, mostly beyond the cingulate sulcus, while C14 is centred (-5, 43, 28) at the medial aspect of the superior frontal gyrus, dorsal to the cingulate sulcus. While these three clusters share many connectivity features with C05, they all have more pronounced FC with the medial parietal cortex (posterior cingulate gyrus and precuneus) and extensive connections with the dorsal and lateral PFC. Both these features are uniquely ascribed to area 32 in macaque tracing studies (Yeterian et al., 2012; Pandya et al., 1981). Similar coupling patterns have also been observed in a human resting-state FC study for seeds placed on the perigenual paracingulate cortex (seeds s5-7 in Margulies et al., 2007; see Cole et al., 2009 for issues of cross-species comparisons in ACC). While C19, C10 and C14, and to some extend also C05, include subdivisions of cytoarchitectonic area 32, with more similar cortical FC profiles, their subcortical connectivity differs. Clusters C05, C19 and C10 are all connected with the amygdala, the hippocampus, the thalamus (both the mediodorsal and the pulvinar thalamic nuclei), the hypothalamus and the ventral and dorsal striatum. However, only C19 is coupled with the periaqueductal gray. In addition, while coupling with amygdala is relatively strong for C05 and C19, it is only weak for C10. With respect to the striatum, all four clusters show coupling with its three major subdivisions, but for intermediate clusters (C19, C10) it is relatively stronger with the ventral striatum, whereas for the clusters at the end points (C05,C14) the coupling peaks in the caudate nucleus. The most caudal cluster C14 differs from the other three in having no connections with the amygdala, the hippocampus or the pulvinar thalamus.

Cluster C12.

On the perigenual cingulate gyrus, caudal to C19, we found cluster C12, with its COM located just anterior to the genu of the corpus callosum COM (-4, 42, 12). This location corresponds to the caudal part of area 24 proper in the four region cingulate model (Palomero-Gallagher et al., 2008). In line with its spatial position, C12's FC profile corresponds to that of area 24 in the monkey (Vogt and Pandya, 1987; Pandya et al., 1981; Morecraft et al., 2012). This profile is characterized by prominent differences, compared to the connectivity of more rostral areas (our C05, C19 ) in the connection with the temporal pole (going dorsally on the cingulate, connectivity becomes more selective and then disappears), the OFC (C05 and C19 are connected with the medial and the caudolateral OFC while C12 is connected only to caudal OFC), the DLPFC (lateral connectivity extends progressively more posterior towards premotor areas and in the superior frontal sulcus), the cingulate (C12 is the only ACC cluster that connects with the entire cingulate) and the frontal operculum (ventral cluster C05 connects mainly with the ventral operculum while connectivity for dorsal C12 extends towards the precentral sulcus). Subcortically, both caudal clusters C12 and C19 show FC with the periaqueductal gray, contrary to C05. Moreover, whereas C12 shares with the more rostral C05 and C19 connectivity with the ventral striatum and the caudate, it shows stronger coupling with the putamen. Contrary to C05 and C19, C12 has only weak coupling with the hypothalamus, but stronger coupling than these clusters with the mediodorsal thalamic nucleus. Lastly, it has weaker connectivity to the amygdala and the hippocampus compared to the other two clusters.

Cluster C13.

As already mentioned, Cluster C13, located caudally to C12 on the cingulate gyrus, presented a discontinuity with the more rostral clusters C12 (area 24 proper) and C14 (32d) dorsally as well as the rest of the clusters on the medial wall. Both spatially and functionally C13 seems to correspond to the most rostral motor cingulate region, mid-cingulate area a24' (Vogt et al., 1995). This is reflected in its FC profile, which is no longer characteristic of the DMN; rather, C13’s FC on the medial wall is confined to the midcingulate region, while there is also strong coupling with the middle frontal gyrus laterally as well as the occipital cortex. C13 shows connections with areas from the task-positive network, the ventral attention and visual networks (Duncan and Owen, 2001; Fox et al., 2006; Corbetta et al., 2008; Stiers et al., 2010; Yeo et al., 2011) consistent with what is seen in the macaque for caudal 24-aMCC (Pandya et al., 1981; Vogt and Pandya, 1987). C13 is significantly coupled with the entire mid-cingulate cortex and preSMA, and on the lateral side with dorsolateral PFC (areas 9/46d, 46 and 44) and the dorsal anterior and middle insula. This pattern confirms previous findings in human resting state FC studies with seeds in the aMCC (Habas, 2010; Hoffstaedter et al., 2014) and is in line with the putative involvement of mid-cingulate cortex in motor preparation/implementation and error monitoring (Hoffstaedter et al., 2014). With respect to our subcortical ROIs, C13 is strongly connected with the mediodorsal thalamus, and moderately with the pulvinar. Further, it is strongly coupled with the amygdala and the dorsal and ventral striatum and moderately with the hypothalamus.

Cluster C09, and C11.

Finally, on the dorsal boundary of our medial patch we have identified clusters C09 and C11 which appear to be the spatial and connectional homologues of medial frontal gyrus areas 10 and 9, respectively (Petrides and Pandya, 1994; Rajkowska and Goldman-Rakic, 1995). C09 occupies the anterior tip of the superior frontal gyrus (COM -11, 65, 6) and has a connectivity fingerprint characteristic of area 10 in tracing studies (Yeterian et al., 2012; Petrides and Pandya, 2012), including significant coupling with the medial parietal and the entire anterior medial frontal cortex. On the lateral side C09 has widespread connectivity with the superior frontal gyrus, the ventral inferior frontal and angular gyrus, and weak connections with the temporal pole and middle temporal gyrus. On the orbital side it is functionally coupled with medial, caudal and lateral orbital areas. The functional coupling of C11 (COM -11, 59, 26) differs from C09 in that on the medial side it has a focus on the anterior aspect of the posterior cingulate gyrus (area 23/p24' in Vogt's model) and on the pregenual ACC. On the lateral side, C11 shows less connectivity with the anterior middle frontal gyrus, and extensive connectivity with the anterior part of the temporal pole. On the orbital side we found evidence of C11 being coupled only with the orbital proisocortex. These characteristics have been described for area 9 in the macaque (Yeterian et al., 2012; Petrides and Pandya, 2012). Subcortically, C11 differs from C09 in that it has a profile strikingly similar to that of our insular/opercular clusters C08 and C07, being strongly connected to the hypothalamus, the ventral and dorsal striatum, the putamen, amygdala, hippocampus and the thalamus. On the other hand, C09 is also strongly connected to the hypothalamus but further has been found coupled only with the ventral striatum and the caudate.

**REFERENCES**

Amaral DG, Price JL. 1984. Amygdalo-cortical projections in the monkey (Macaca fascicularis). J Comp Neurol. 230:465-496.

Amiez C, Petrides M. 2014. Neuroimaging evidence of the anatomo-functional organization of the human cingulate motor areas. Cereb Cortex. 24:563–578.

Augustine JR. 1996. Circuitry and functional aspects of the insular lobe in primates including humans. Brain Res Rev. 22:229–244.

Baxter MG, Parker A, Lindner CC, Izquierdo AD, Murray EA. 2000. Control of response selection by reinforcer value requires interaction of amygdala and orbital prefrontal cortex. J Neurosci. 20:4311-9.

Beck E. 1949. A cytoarchitectural investigation into the boundaries of cortical areas 13 and 14 in the human brain. J Anat. 83:147–157.

Beckmann M, Johansen-Berg H, Rushworth MFS. 2009. Connectivity-based parcellation of human cingulate cortex and its relation to functional specialization. J Neurosci. 29:1175–1190.

Bludau S, Eickhoff SB, Mohlberg H, Caspers S, Laird a R, Fox PT, Schleicher a, Zilles K, Amunts K. 2014. Cytoarchitecture, probability maps and functions of the human frontal pole. Neuroimage. 93: 260- 275.

Buckner RL, Andrews-Hanna JR, Schacter DL. 2008. The brain’s default network: anatomy, function, and relevance to disease. Ann N Y Acad Sci. 1124:1–38.

Carmichael ST, Price JL. 1994. Architectonic subdivision of the orbital and medial prefrontal cortex in the macaque monkey. J Comp Neurol. 346:366–402.

Carmichael ST, Price JL. 1996. Connectional networks within the orbital and medial prefrontal cortex of macaque monkeys. J Comp Neurol. 371:179–207.

Catani M, Dell’acqua F, Vergani F, Malik F, Hodge H, Roy P, Valabregue R, Thiebaut de Schotten M. 2012. Short frontal lobe connections of the human brain. Cortex. 48:273–291.

Cauda F, D’Agata F, Sacco K, Duca S, Geminiani G, Vercelli A. 2011. Functional connectivity of the insula in the resting brain. Neuroimage. 55:8–23.

Cavada C, Compañy T, Tejedor J, Cruz-Rizzolo RJ, Reinoso-Suárez F. 2000. The anatomical connections of the macaque monkey orbitofrontal cortex. A review. Cereb Cortex. 10:220–242.

Cerliani L, Thomas RM, Jbabdi S, Siero JCW, Nanetti L, Crippa A, Gazzola V, D’Arceuil H, Keysers C. 2012. Probabilistic tractography recovers a rostrocaudal trajectory of connectivity variability in the human insular cortex. Hum Brain Mapp. 33:2005–2034.

Chikama M, McFarland NR, Amaral DG, Haber SN. 1997. Insular cortical projections to functional regions of the striatum correlate with cortical cytoarchitectonic organization in the primate. J Neurosci. 17:9686–9705.

Chumbley JR, Friston KJ. 2009. False discovery rate revisited: FDR and topological inference using Gaussian random fields. Neuroimage. 44:62–70.

Cole MW, Yeung N, Freiwald WA, Botvinick M. 2009. Cingulate cortex: diverging data from humans and monkeys. Trends Neurosci. 32:566–574.

Corbetta M, Patel G, Shulman GL. 2008. The reorienting system of the human brain: from environment to theory of mind. Neuron. 58:306 –324.

Deen B, Pitskel NB, Pelphrey KA. 2011. Three systems of insular functional connectivity identified with cluster analysis. Cereb Cortex. 21:1498–1506.

Devinsky O, D’Esposito M. 2004. Neurology of cognitive and behavioral disorders. New York: Oxford University Press.

Drevets WC, Price JL, Furey ML. 2008. Brain structural and functional abnormalities in mood disorders: implications for neurocircuitry models of depression. Brain Struct Funct. 213:93–118.

Duncan J, Owen AM. 2000. Common regions of the human frontal lobe recruited by diverse cognitive demands. Trends Neurosci. 23:475– 483.

Ferry AT, Öngür D, An X, Price JL. 2000. Prefrontal cortical projections to the striatum in macaque monkeys: evidence for an organization related to prefrontal networks. J Comp Neurol 425: 447–470.

Flynn FG, Benson DF, Ardilas A. 1999. Anatomy of the insula – functional and clinical correlates. Aphasiology. 13:55–78.

Ford A, McGregor KM, Case K, Crosson B, White KD. 2010. Structural connectivity of Broca’s area and medial frontal cortex. Neuroimage. 52:1230–1237.

Fox MD, Corbetta M, Snyder AZ, Vincent JL, Raichle ME. 2006. Spontaneous neuronal activity distinguishes human dorsal and ventral attention systems. Proc Natl Acad Sci U S A. 103:10046 –10051.

Gallay DS, Gallay MN, Jeanmonod D, Rouiller EM, Morel A. 2012. The insula of Reil revisited: multiarchitectonic organization in macaque monkeys. Cereb Cortex. 22:175–190.

Garrett EA, DeLong MR, Strick PL. 1986. Parallel organization of functionally segregated circuits linking basal ganglia and cortex. Ann Rev Neurosci. 9:357-81.

Ghashghaei HT, Hilgetag CC, Barbas H. 2007. Sequence of information processing for emotions based on the anatomic dialogue between prefrontal cortex and amygdala. Neuroimage. 34:905–923.

Gilbert SJ, Spengler S, Simons JS, Steele JD, Lawrie SM, Frith CD, Burgess PW. 2006. Functional specialization within rostral prefrontal cortex (area 10): a meta-analysis. J Cogn Neurosci. 18:932–948.

Goebel R, Esposito F, Formisano E. 2006. Analysis of functional image analysis contest (FIAC) data with brainvoyager QX: From single-subject to cortically aligned group general linear model analysis and self-organizing group independent component analysis. Hum Brain Mapp. 27:392–401.

Goulas A, Uylings HBM, Stiers P. 2012. Unravelling the intrinsic functional organization of the human lateral frontal cortex: a parcellation scheme based on resting state fMRI. J Neurosci. 32:10238–10252.

Groman SM, James AS, Seu E, Crawford MA, Harpster SN, Jentsch JD. 2013. Monoamine levels within the orbitofrontal cortex and putamen interact to predict reversal learning performance. Biol Psychiatry. 73:756–762.

Guimerá R, Amaral LAN. 2005. Functional cartography of complex metabolic networks. Nature. 433:895–900.

Habas C. 2010. Functional connectivity of the human rostral and caudal cingulate motor areas in the brain resting state at 3T. Neuroradiology. 52:47–59.

Haber SN, Kunishio K, Mizobuchi M. 1995. The Orbital and Medial Prefrontal Circuit Through the Primate Basal Ganglia. J Neurosci. 75:4851–4867.

Hof PR, Mufson EJ, Morrison JH. 1995. Human orbitofrontal cortex: cytoarchitecture and quantitative immunohistochemical parcellation. J Comp Neurol. 359:48–68.

Hoffstaedter F, Grefkes C, Caspers S, Roski C, Palomero-Gallagher N, Laird AR, Fox PT, Eickhoff SB. 2014. The role of anterior midcingulate cortex in cognitive motor control: Evidence from functional connectivity analyses. Hum Brain Mapp. 35:2741-2753.

Johansen-Berg H, Gutman DA, Behrens TEJ, Matthews PM, Rushworth MFS, Katz E, Lozano AM, Mayberg HS. 2008. Anatomical connectivity of the subgenual cingulate region targeted with deep brain stimulation for treatment-resistant depression. Cereb Cortex. 18:1374–1383.

Kahnt T, Chang LJ, Park SQ, Heinzle J, Haynes J-D. 2012. Connectivity-based parcellation of the human orbitofrontal cortex. J Neurosci. 32:6240–6250.

Kelly C, Uddin LQ, Shehzad Z, Margulies DS, Castellanos FX, Milham MP, Petrides M. 2010. Broca’s region: linking human brain functional connectivity data and non-human primate tracing anatomy studies. Eur J Neurosci. 32:383–398.

Koski R, Paus T. 2000. Functional connectivity of the anterior cingulate cortex within the human frontal lobe : a brain-mapping meta-analysis. Exp Brain Res. 12:55–65.

Kringelbach ML, Rolls ET. 2004. The functional neuroanatomy of the human orbitofrontal cortex: evidence from neuroimaging and neuropsychology. Prog Neurobiol. 72:341–372.

Kringelbach ML. 2005. The human orbitofrontal cortex: linking reward to hedonic experience. Nat Rev Neurosci. 6:691–702.

Kurth F, Zilles K, Fox PT, Laird AR, Eickhoff SB. 2010. A link between the systems: functional differentiation and integration within the human insula revealed by meta-analysis. Brain Struct Funct. 214:519–534.

Liu H, Qin W, Li W, Fan L, Wang J, Jiang T, Yu C. 2013. Connectivity-based parcellation of the human frontal pole with diffusion tensor imaging. J Neurosci. 33:6782–6790.

Mackey S, Petrides M. 2009. Architectonic mapping of the medial region of the human orbitofrontal cortex by density profiles. Neuroscience. 159:1089–1107.

Margulies DS, Petrides M. 2013. Distinct parietal and temporal connectivity profiles of ventrolateral frontal areas involved in language production. J Neurosci. 33:16846–16852.

Margulies DS, Kelly AMC, Uddin LQ, Biswal BB, Castellanos FX, Milham MP. 2007. Mapping the functional connectivity of anterior cingulate cortex. Neuroimage. 37:579–588.

Margulies DS, Vincent JL, Kelly C, Lohmann G, Uddin LQ, Biswal BB, Villringer A, Castellanos FX, Milham MP, Petrides M. 2009. Precuneus shares intrinsic functional architecture in humans and monkeys. Proc Natl Acad Sci USA. 106:20069 –20074.

Medford N, Critchley HD. 2010. Conjoint activity of anterior insular and anterior cingulate cortex: awareness and response. Brain Struct Funct. 214:535–549.

Mesulam M-M, Mufson EJ. 1982. Insula of the old world monkey. III: Efferent cortical output and comments on function. J Comp Neurol. 212:38-52.

Moayedi M, Salomons T V, Dunlop K a M, Downar J, Davis KD. 2014. Connectivity-based parcellation of the human frontal polar cortex. Brain Struct Funct. DOI 10.1007/s00429-014-0809-6.

Morecraft RJ, Geula C, Mesulam M-M. 1992. Cytoarchitecture and neural afferents of orbitofrontal cortex in the brain of the monkey. J Comp Neurol. 323:341-358.

Morecraft RJ, Stilwell-Morecraft KS, Cipolloni PB, Ge J, McNeal DW, Pandya DN. 2012. Cytoarchitecture and cortical connections of the anterior cingulate and adjacent somatomotor fields in the rhesus monkey. Brain Res Bull. 87:457-497.

Morris JS, Dolan RJ. 2001. Involvement of human amygdala and orbitofrontal cortex in hunger-enhanced memory for food stimuli. J Neurosci. 21:5304-10.

Mufson EJ, Mesulam M-M. 1982. Insula of the old world monkey. II: Afferent cortical input and comments on the claustrum. J Comp Neurol. 212:23-37.

Muranishi M, Inokawa H, Yamada H, Ueda Y, Matsumoto N, Nakagawa M, Kimura M.2011. Inactivation of the putamen selectively impairs reward history-based action selection. Exp Brain Res. 209:235–246.

Nelson SM, Dosenbach NUF, Cohen AL, Wheeler ME, Schlaggar BL, Petersen SE. 2010. Role of the anterior insula in task-level control and focal attention. Brain Struct Funct. 214:669–680.

Neubert F-X, Mars RB, Thomas AG, Sallet J, Rushworth MFS. 2014. Comparison of Human Ventral Frontal Cortex Areas for Cognitive Control and Language with Areas in Monkey Frontal Cortex. Neuron. 81:700–713.

Ongür D, Price JL. 2000. The organization of networks within the orbital and medial prefrontal cortex of rats, monkeys and humans. Cereb Cortex. 10:206–219.

Ongür D, Ferry AT, Price JL. 2003. Architectonic subdivision of the human orbital and medial prefrontal cortex. J Comp Neurol. 460:425–449.

Palomero-Gallagher N, Mohlberg H, Zilles K, Vogt BA. 2008. Cytology and receptor architecture of human anterior cingulate cortex. J Comp Neurol. 508:906–926.

Palomero-Gallagher N, Zilles K, Schleicher A, Vogt BA. 2013. Cyto- and receptor architecture of area 32 in human and macaque brains. J Comp Neurol. 521:3272–3286.

Pandya DN, Van Hoesen GW, Mesulam M-M. 1981. Efferent connections of the cingulate gyrus in the rhesus monkey. Exp Brain Res. 9:319–330.

Petrides M, Pandya DN. 1994. Comparative architectonic analysis of the human and the macaque frontal cortex. In: Handbook of Neuropsychology (Boller F, Grafman J, ed), pp17-58. Amsterdam: Elsevier.

Petrides M, Pandya DN. 2012. The frontal cortex. In: The human nervous system (Mai JK, Paxinos G, ed), pp988-1011. Elsevier AP.

Rajkowska G, Goldman-Rakic PS. 1995. Cytoarchitectonic definition of prefrontal areas in the normal human cortex. I. Remapping of areas 9 and 46 using quantitative criteria. Cereb Cortex. 5:307–322.

Rolls ET, Grabenhorst F. 2008. The orbitofrontal cortex and beyond: from affect to decision-making. Prog Neurobiol. 86:216–244.

Rorden C, Brett M. 2000. Stereotaxic display of brain lesions. Behav Neurol. 12:191–200.

Ross RS, Lopresti ML, Schon K, Stern CE. 2013. Role of the hippocampus and orbitofrontal cortex during the disambiguation of social cues in working memory. Cogn Affect Behav Neurosci. 13:900–915.

Rudebeck PH, Murray EA. 2011. Balkanizing the primate orbitofrontal cortex: distinct subregions for comparing and contrasting values. Ann N Y Acad Sci. 1239:1–13.

Rushworth MFS, Noonan MP, Boorman ED, Walton ME, Behrens TE. 2011. Frontal cortex and reward-guided learning and decision-making. Neuron. 70:1054–1069.

Rushworth MFS, Mars RB, Sallet J. 2013. Are there specialized circuits for social cognition and are they unique to humans? Curr Opin Neurobiol. 23:436–442.

Sallet J, Mars RB, Noonan MP, Neubert F-X, Jbabdi S, O’Reilly JX, Filippini N, Thomas AG, Rushworth MF. 2013. The organization of dorsal frontal cortex in humans and macaques. J Neurosci. 33:12255–12274.

Seeley WW, Menon V, Schatzberg AF, Keller J, Glover GH, Kenna H, Reiss AL, Greicius MD. 2007. Dissociable intrinsic connectivity networks for salience processing and executive control. J Neurosci. 27:2349–2356.

Selemon LD, Goldman-Rakic PS. 1985. Longitudinal topography and interdigitation of corticostriatal projections in the rhesus monkey. J Neurosci. 5:776-794.

Stiers P, Mennes M, Sunaert S. 2010. Distributed task coding throughout the multiple demand network of the human frontal-insular cortex. Neuroimage. 52:252–262.

Taylor KS, Seminowicz DA, Davis KD. 2009. Two systems of resting state connectivity between the insula and cingulate cortex. Hum Brain Mapp. 30:2731–2745.

Timbie C, Barbas H. 2014. Specialized pathways from the primate amygdala to posterior orbitofrontal cortex. J Neurosci. 34:8106–8118.

Tziortzi AC, Searle GE, Tzimopoulou S, Salinas C, Beaver JD, Jenkinson M, Laruelle M, Rabiner EA, Gunn RN. 2011. Imaging dopamine receptors in humans with [11C]-(+)-PHNO: dissection of D3 signal and anatomy. Neuroimage. 54:264–277.

Uylings HBM, Sanz-Arigita EJ, de Vos K, Pool CW, Evers P, Rajkowska G. 2010. 3-D cytoarchitectonic parcellation of human orbitofrontal cortex correlation with postmortem MRI. Psychiatry Research: Neuroimaging. 183:1–20.

Van Eijndhoven P, Van Wingen G, Katzenbauer M, Groen W, Tepest R, Fernández G, Buitelaar J, Tendolkar I. 2013. Paralimbic Cortical Thickness in First-Episode Depression: Evidence for Trait-Related Differences in Mood Regulation. Am J Psychiat. 170:1477–1486.

Vogt BA. 2005. Pain and emotion interactions in subregions of the cingulate gyrus. Nat Rev Neurosci. 6:533–544.

Vogt BA, Hof PR, Zilles K, Vogt LJ, Herold C, Palomero-Gallagher N. 2013. Cingulate area 32 homologies in mouse, rat, macaque and human: cytoarchitecture and receptor architecture. J Comp Neurol. 521:4189–4204.

Vogt BA, Nimchinsky EA, Vogt LJ, Hof PR. 1995. Human cingulate cortex: surface features, flat maps, and cytoarchitecture. J Comp Neurol. 359:490–506.

Vogt BA, Pandya DN. 1987. Cingulate cortex of the rhesus monkey. II: Cortical afferents. J Comp Neurol. 262:271-289.

Vogt BA, Vogt L, Farber NB, Bush G. 2005. Architecture and neurocytology of monkey cingulate gyrus. J Comp Neurol. 485:218–239.

Yeo BT, Krienen FM, Sepulcre J, Sabuncu MR, Lashkari D, Hollinshead M, Roffman JL, Smoller JW, Zöllei L, Polimeni JR, Fischl B, Liu H, Buckner RL. 2011. The organization of the human cerebral cortex estimated by functional connectivity. J Neurophysiol. 106:1125–1165.

Yeterian EH, Pandya DN, Tomaiuolo F, Petrides M. 2012. The cortical connectivity of the prefrontal cortex in the monkey brain. Cortex. 48:58–81.

Yu C, Zhou Y, Liu Y, Jiang T, Dong H, Zhang Y, Walter M. 2011. Functional segregation of the human cingulate cortex is confirmed by functional connectivity based neuroanatomical parcellation. Neuroimage. 54:2571–2581.

Zou Q, Long X, Zuo X, Yan C, Zhu C, Yang Y, Liu D, He Y, Zang Y. 2009. Functional connectivity between the thalamus and visual cortex under eyes closed and eyes open conditions: a resting-state fMRI study. Hum Brain Mapp. 30:3066–3078.

**TABLES**

| **Table 2. Putative anatomical substrates for the left hemisphere clusters.**  **Cluster numbers correspond to numbering of parcellation map (paper, Figure 1). Candidate anatomical substrates are assigned for most clusters; see paper, Figure 5 & 6 and cited papers for the cytoarchitectonic maps to which nomenclature refers to.**   |  | | --- |  | **Cluster Published Nomenclatures** |  |  |  | | --- | --- | --- | --- | | | C01 | **11, 47m^1^, 47m^2^** (Uylings et al., 2010)  **14** (Petrides and Pandya 1994)  **14r** (Ongür et al., 2003; Mackay and Petrides, 2010) | |  | | | --- | --- | --- | --- | --- | | C02 | **47m^3^** (Uylings et al., 2010)  **13** (Petrides and Pandya 1994) |  |  | | | C03 | **47l^1^, 47l^2^** (Uylings et al., 2010)  **47/12** (Petrides and Pandya 1994) |  |  | | | C04 | **10** (Petrides and Pandya 1994) |  |  | | | C05 | **25, 24** (Petrides and Pandya 1994)  **s24, s32** (Palomero-Gallagher et al., 2008; Vogt et al., 2003;  Vogt and Palomero-Gallagher, 2012) | | | | | C06 | **47/12, 10** (Petrides and Pandya 1994) |  |  | | | C07 | **47l^2^** (Uylings et al., 2010)  **45A** (Petrides and Pandya 1994) |  |  | | | C08 | **Iai** (Ongür et al., 2003) |  |  | | | C09 | **10** (Petrides and Pandya 1994) |  |  | | | C10 | **p32** (Palomero-Gallagher et al., 2013) |  |  | | | C11 | **9** (Petrides and Pandya 1994) |  |  | | | C12 | **p24** (Palomero-Gallagher et al., 2013) |  |  | | | C13 | **a24'** (Vogt et al., 1995) |  |  | | | C14 | **d32** (Palomero-Gallagher et al., 2008; Vogt et al., 2003; Vogt and Palomero-Gallagher, 2012) |  | |  | | C15 | **47m^3^** (Uylings et al., 2010) **11** (Petrides and Pandya 1994) |  |  | | | C16 | **11, 47m^1^, 47m^2^** (Uylings et al., 2010)  **14** (Petrides and Pandya 1994)  **14c** (Ongür et al., 2003; Mackay and Petrides, 2010) | |  | | | C17 | **BA10** (Brodmann, 1909) |  |  | | | C18 | **10** (Petrides and Pandya 1994)  **10p** (Ongür et al., 2003) |  |  | | | C19 | **p32** (Palomero-Gallagher et al., 2008; Vogt et al., 2003; Vogt and Palomero-Gallagher, 2012) |  |  | | |  |  |  | |
| --- | --- | --- | --- | --- | --- | --- | --- | --- | --- | --- | --- | --- | --- | --- | --- | --- | --- | --- | --- | --- | --- | --- | --- | --- | --- | --- | --- | --- | --- | --- | --- | --- | --- | --- | --- | --- | --- | --- | --- | --- | --- | --- | --- | --- | --- | --- | --- | --- | --- | --- | --- | --- | --- | --- | --- | --- | --- | --- | --- | --- | --- | --- | --- | --- | --- | --- | --- | --- | --- | --- | --- | --- | --- | --- | --- | --- | --- | --- | --- | --- | --- | --- | --- | --- | --- | --- | --- | --- | --- | --- | --- | --- | --- | --- | --- | --- | --- | --- | --- | --- | --- | --- | --- | --- |

**FIGURES.**

**Figure S1:**

Whole-brain cortical and subcortical functional connectivity profiles of the orbital, ventrolateral PFC and insulo-opercular clusters. Bright colors in the cortical profiles represent higher *t* values (voxel-level threshold *p*=.01; cluster-level threshold *p*=.005). Radar plots illustrate separately the FC of each cluster with the amygdala (***AMG***), hippocampus (***HPC***), thalamus (***THL***), mediodorsal (***THL MD***) and pulvinar (***THL PUL***) thalamic nuclei, hypothalamus (***HTH***), periaqueductal gray (***PAG***), ventral striatum (***VST***), caudate (***CDT***) and putamen (***PTM***).


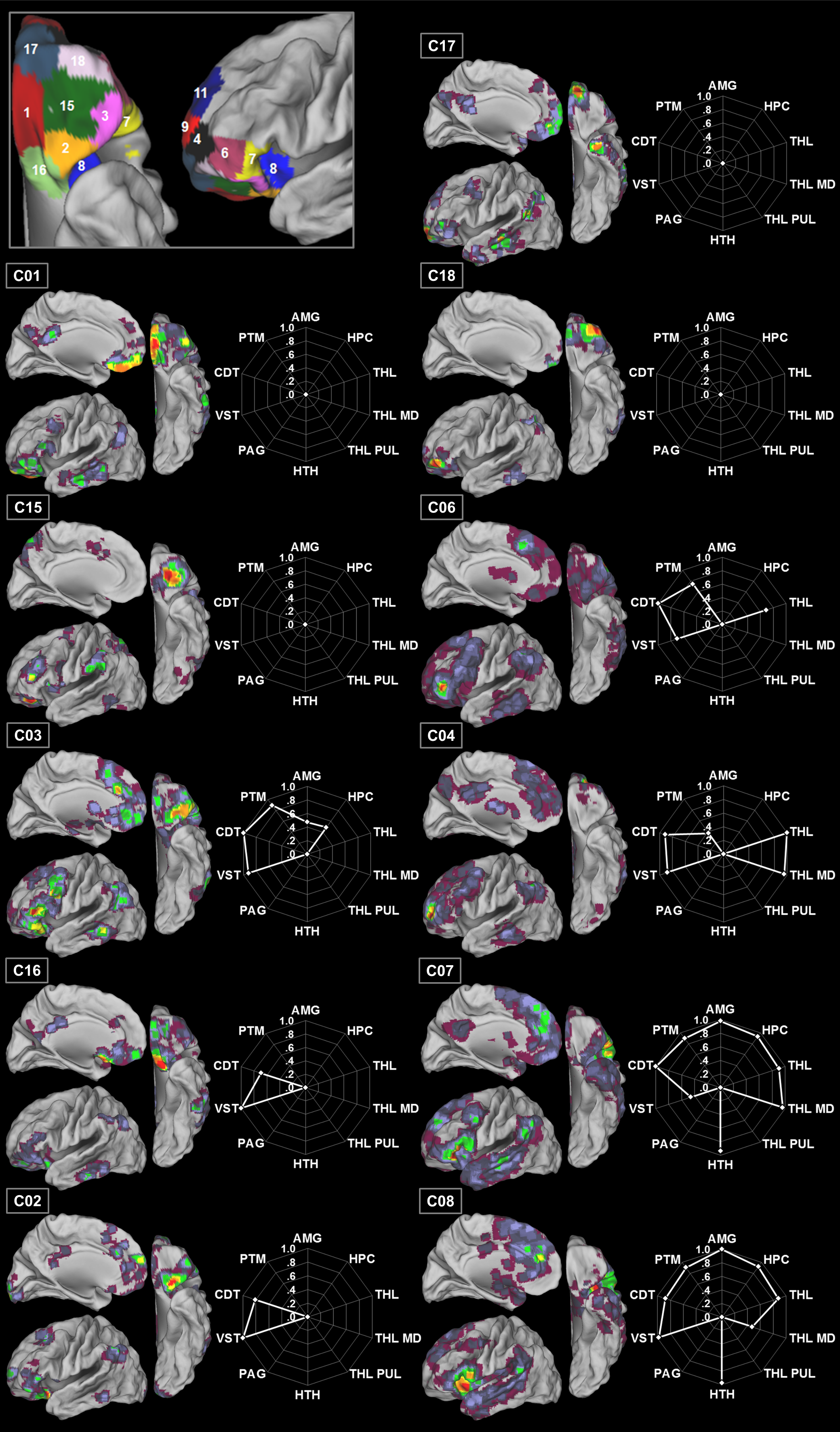


**Figure S2:**

Whole-brain cortical and subcortical functional connectivity profiles of the medial clusters. Bright colors in the cortical profiles represent higher *t* values (voxel-level threshold *p*=.01; cluster-level threshold *p*=.005). Radar plots illustrate separately the FC of each cluster with the amygdala (***AMG***), hippocampus (***HPC***), thalamus (***THL***), mediodorsal (***THL MD***) and pulvinar (***THL PUL***) thalamic nuclei, hypothalamus (***HTH***), periaqueductal gray (***PAG***), ventral striatum (***VST***), caudate (***CDT***) and putamen (***PTM***).


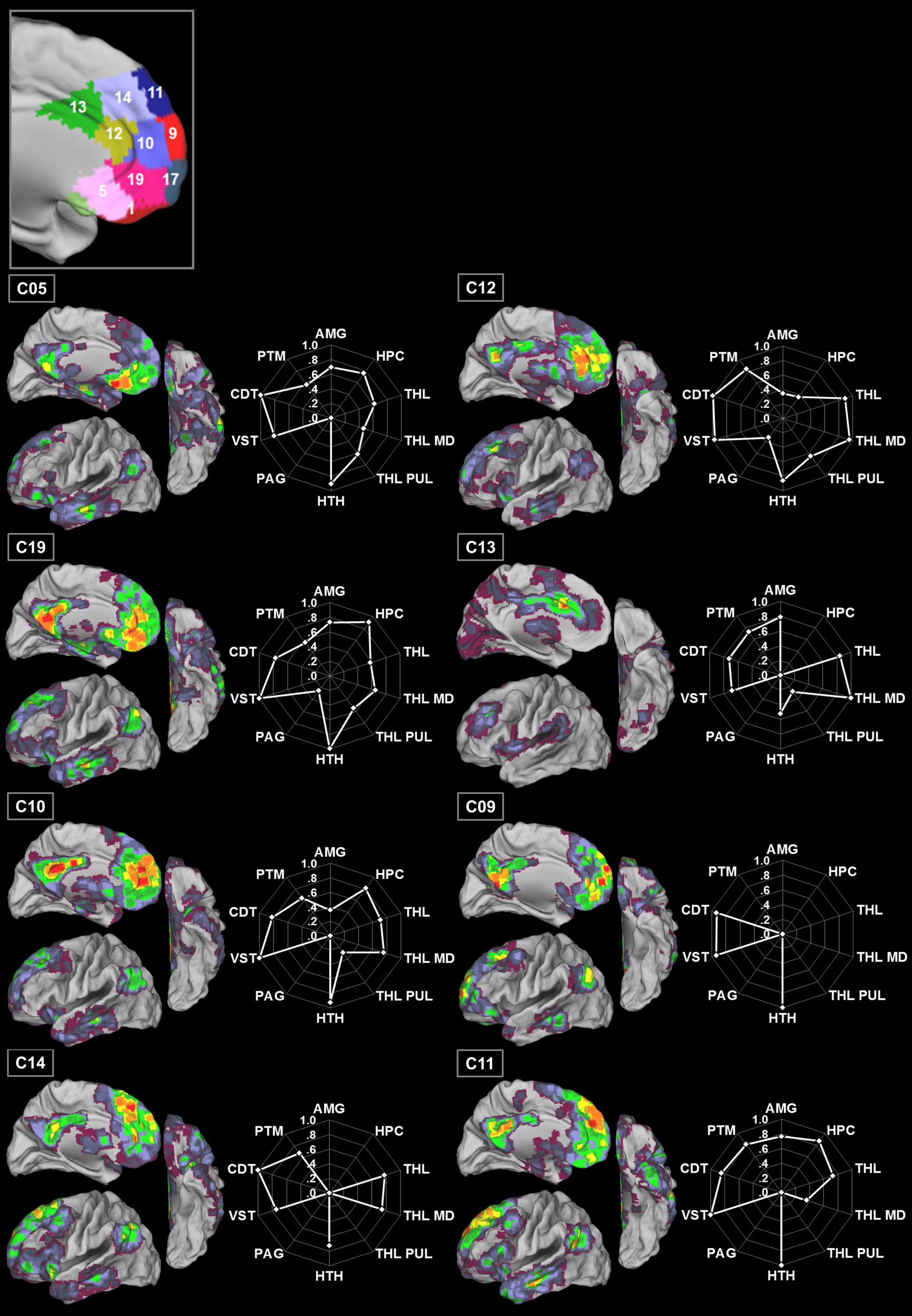

Supplement: Supplementary file 1 — Supplementary material 1 (DOC 8016 KB) [file 429_2017_1378_MOESM1_ESM.doc]
